# New Peptides from The Marine-Derived Fungi *Aspergillus allahabadii* and *Aspergillus ochraceopetaliformis*

**DOI:** 10.3390/md17090488

**Published:** 2019-08-21

**Authors:** Ji-Yeon Hwang, Jung-Ho Lee, Sung Chul Park, Jayho Lee, Dong-Chan Oh, Ki-Bong Oh, Jongheon Shin

**Affiliations:** 1 Natural Products Research Institute, College of Pharmacy, Seoul National University, San 56-1, Sillim, Gwanak, Seoul 151-742, Korea; 2 Department of Agricultural Biotechnology, College of Agriculture and Life Science, Seoul National University, San 56-1, Sillim, Gwanak, Seoul 151-921, Korea

**Keywords:** peptides, marine-derived fungi, *Aspergillus allahabadii*, *Aspergillus ochraceopetaliformis*, isocitrate lyase, sortase A

## Abstract

Four new peptides were isolated from the culture broths of the marine-derived fungi *Aspergillus* *allahabadii* and *A.* *ochraceopetaliformis.* Based on the results of chemical and spectroscopic analyses, two compounds (**1** and **2**) from *A.* *allahabadii* were determined to be cyclopentapeptides, while those from *A.* *ochraceopetaliformis* were a structurally-related cyclodepsihexapeptide (**3**) and its linear analog (**4**). In addition to the presence of a D-amino acid residue, the almost reversed sequence of peptides in **3** and **4,** relative to those of the **1** and **2**, is notable. These new compounds exhibited moderate inhibition against the enzyme sortase A as well as a weak inhibition against isocitrate lyase (**2**).

## 1. Introduction

Recently, fungal peptides have attracted significant interest due to their beneficial effects in promoting health and reducing disease [1]. Indeed, bioactive peptides and peptide-bearing compounds have been used as lead compounds in drug development. One representative example is the lipopeptide pneumocandin B_0_ from *Glarea lozoyensis*, which was approved as the antifungal drug caspofungin acetate (CANCIDAS^®^) [2,3]. Another notable example is the diketopiperazine halimide from a marine-derived *Aspergillus* sp., as its synthetic analog plinabulin is currently in clinical trials as a new anticancer agent [4].

As found from their counterparts from terrestrial environments, peptides are among the major groups of marine fungal natural products [5,6,7]. Considering the explosive progress in chemical investigation of marine fungi, demonstrated by the isolation of more than 3500 novel compounds, the potential of these organisms as the prolific sources of bioactive peptides is expected to continually increase [8,9,10,11,12,13]. Among the marine fungi, peptides and structurally-related compounds have mainly been isolated from the genera *Acremonium*, *Aspergillus*, *Fusarium*, and *Penicillium* [3]. In particular, the ascomycete genus *Aspergillus* is well known producer of small to medium-sized cyclic tri- to penta-peptides [3].

During the course of a search for new compounds from marine-derived fungi, we isolated strains of *Aspergillus allahabadii* and *A. ochraceopetaliformis* from underwater sediment obtained off the coast of Jaeju-do (island), Korea. The LC-UV and LC-ESI-MS analyses of the culture broths were conducted and compared with our in-house spectrum library of fungal extracts. This approach indicated the presence of four putatively unknown compounds, prompting an extensive chemical investigation. A large-scale cultivation followed by solvent-partitioning and chromatographic separation afforded four peptides. Here, we report the structures of two cyclopentapeptides (**1** and **2**), a related cyclohexadepsipeptide (**3**) and its linear analog (**4**). These compounds moderately inhibited microbe-derived enzymes, sortase A (SrtA) and isocitrate lyase (ICL, only **2**).

## 2. Results and Discussion

The molecular formula of compound **1** was deduced to be C_33_H_43_N_5_O_6_ with 15 degrees of unsaturation by HRFABMS analysis ([M + H]^+^ *m/z* 606.3296, calcd for C_33_H_44_N_5_O_6,_ 606.3292). The ^13^C NMR data of this compound showed signals at δ_C_ 171.2, 170.6, 170.4, 168.9, and 168.2 for carbonyl carbons (Table 1). In conjunction with the strong absorption band at 1649 cm^−1^ in the IR spectrum, these carbons were thought to be amide carbons, indicating a peptidic compound. Signals for the corresponding exchangeable peptide NH protons were observed at δ_H_ 8.29, 7.27, and 6.96 in the ^1^H NMR spectra. Other conspicuous features in the NMR data were several de-shielded carbons (δ_C_ 155.7–14.9) and protons (δ_H_ 7.28–6.66), accounting for two aromatic moieties. All the remaining carbon and proton signals were in the shielded region, suggesting the presence of aliphatic amino acids. Thus, compound **1** must possess two rings from the 15 degrees of unsaturation inherent in its formula.

**Figure 1:**
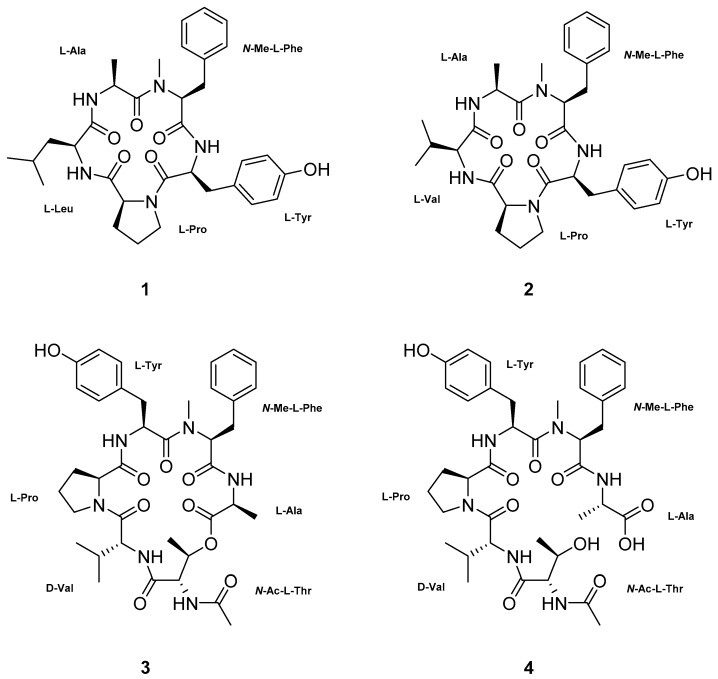
The structures of **1**–**4**.

Given this information, the structure of compound **1** was determined by a stepwise interpretation of the 2-D NMR data. First, based on the results of HSQC analysis, all the protons and their attached carbons were precisely matched. Then, the ^1^H-^1^H COSY data defined several proton spin systems, including two involving exchangeable NH protons: NH-CH-CH_3_ and NH-CH-CH_2_-CH(CH_3_)_2_. In this way, two NH-bearing aliphatic moieties were readily identified as alanine (Ala) and leucine (Leu) (Figure 1). This interpretation was confirmed by the HMBC data, which showed diagnostic intra-residual three-bond correlations with the *β*-carbonyl-*β*-NH protons, allowing the assignment of the carbonyl carbons for each amino acid residue. Thus, the carbonyl carbons at δ_C_ 171.2 and 170.4 were assigned as the amide carbonyls of Ala and Leu, respectively.

The COSY data identified the two aromatic moieties as *p*-hydroxyphenyl and phenyl groups (Tables S2–S5). Starting with the HMBC correlations between the ring protons (δ_H_ 7.04) and a benzylic carbon (δ_C_ 37.3), the tracing of proton–proton and carbon–proton correlations identified a carbonyl (δ_C_ 168.9), an α-methine (δ_C_ 52.6, δ_H_ 4.78) and an NH (δ_H_ 7.27) of a tyrosine (Tyr) residue. Similar 2-D NMR analyses indicated carbonyl (δ_C_ 168.1), α-methine (δ_C_ 61.7, δ_H_ 4.17), and *β*-methylene/benzylic (δ_C_ 33.9, δ_H_ 3.24, and 2.70) groups. However, instead of NH, the presence of an NCH_3_ group (δ_C_ 30.5, δ_H_ 2.61) was confirmed by crucial HMBC correlation of H_3_-NCH_3_/C-α, indicating the presence of an *N*-methylphenylalanine (*N*-MePhe) residue.

**Figure 2:**
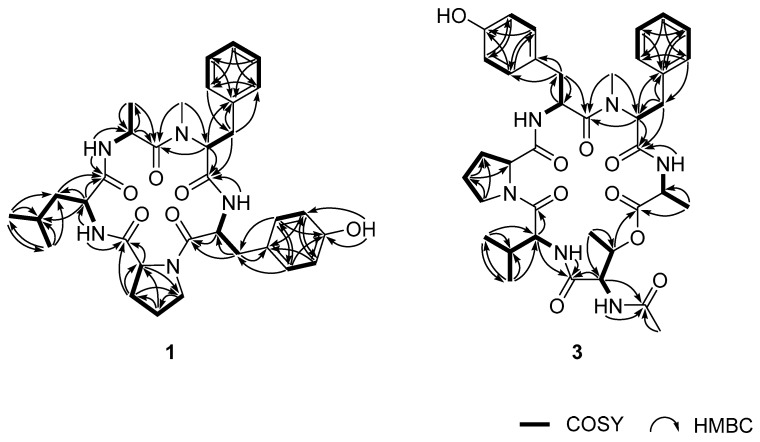
The COSY (bold) and HMBC (arrows) key correlations of compounds **1** and **3**.

For the remaining residue, the HSQC and COSY data identified the linear connectivity of a methine (δ_C_ 60.9, δ_H_ 4.10) and three methylenes (δ_C_ 31.5, δ_H_ 1.95, 1.88; δ_C_ 21.4, δ_H_ 1.77, δ_H_ 1.58; δ_C_ 46.4, δ_H_ 3.47, 3.37). The characteristic chemical shifts of a terminal methine and methylene groups as well as the lack of spin-coupled NH protons were indicative of a proline (Pro) residue, accounting for one of the two rings in the structure. The presence of this residue was confirmed by the HMBC data. The ring moiety of Pro was assigned based on the key correlations of H-α/C-*δ* and CO, H_2_-*β*/C-*δ* and CO, and H_2_-*δ*/C-*γ* (Figure 2).

The assembly of these amino acid residues into a cyclopeptide was accomplished using sequential inter-residue HMBC correlations between the protons and carbons belonging to neighboring amino acid residues (Figure 2). The peptide linkage between Tyr and *N*-MePhe was revealed by the two-bond correlation of NH (Tyr)–CO (*N*-MePhe). The latter amino acid was linked to Ala by the correlations of H_3_-NCH_3_ (*N*-MePhe)–CO (Ala) and H-α (*N*-MePhe)–CO (Ala). Similarly, the correlation of NH (Ala)–CO (Leu) linked these amino acids. Finally, this Leu was bound via a peptide bond to Pro based on the correlation of NH (Leu)–CO (Pro). Although it was not directly proven by the HMBC data, the molecular formula requires the presence of an additional ring, confirming a peptide linkage between N (Pro) and CO (Tyr), since the structure of **1** has 15 degrees of unsaturation. Five are equivalent to five carbonyl groups from the peptide linkage, eight are equivalent to two aromatic groups of Tyr and *N*-MePhe, one is equivalent to a ring from Pro. As no more double bond carbon is left, the only way to satisfy the 15 degrees of unsaturation is by connecting a peptide linkage between N (Pro) and CO (Tyr). 

**Figure 3:**
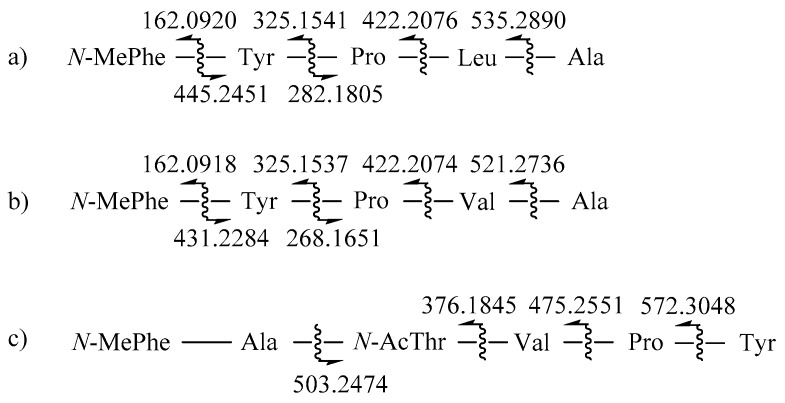
The high-resolution LC/MS-MS fragmentation analysis of compounds **1**–**3** (**a**–**c**).

The structure of **1** derived from the NMR data was confirmed by high-resolution LC-MS/MS analysis, in which all of the peptide bonds between neighboring amino acid residues were sequentially cleaved (Figure 3). Finally, the absolute configurations of all the amino acids were assigned as L by advanced Marfey’s analysis (Figure S32, Table S1). Thus, the structure of compound **1**, designated to be JG002CPA, was determined to be a new cyclopentapeptide. 

The molecular formula of compound **2** was established to be C_32_H_41_N_5_O_6_ by HRFABMS analysis ([M + H]^+^ *m/z* 592.3133, calcd for C_32_H_42_N_5_O_6,_ 592.3135). The NMR data of this compound were very similar to those of **1**, suggesting the same pentacyclopeptide nature. The most noticeable difference in the ^13^C and ^1^H NMR data was the lack of an aliphatic methylene in **2**. A detailed examination of the NMR data revealed that the signals of the carbons and protons of the Leu in **1** were replaced with those of a valine (Val), while the signals from other four residues were the same between of **1** (Table 1). This interpretation was unambiguously confirmed by combined 2-D NMR data, as all the key carbon–proton and proton–proton correlations were found (Figure 2). Subsequently, **2** was shown to have the same amino acid sequence as **1** by LC-MS/MS analysis (Figure 3). The absolute configurations of all amino acids were also assigned as L, identical to **1**, by advanced Marfey’s analysis (Figure S32, Table S1). Thus, the structure of compound **2**, designated to be JG002CPB, was determined to be a cyclopentapeptide structurally closely related to JG002CPA (**1**). Literature studies showed that the most related cyclopeptides to **1** and **2** are cotteslosin A and B, which were previously isolated from the marine-derived fungus *Aspergillus versicolor* [14].

Compound **3** was isolated as an amorphous solid from the culture broth of *A*. *ochraceopetaliformis* and determined to have a formula of C_38_H_50_N_6_O_9_ by HRFABMS analysis ([M + H]^+^ *m/z* 735.3721, calcd for C_38_H_51_N_6_O_9,_ 735.3718). The ^13^C NMR data of this compound showed signals at δ_C_ 173.3, 172.4, 172.0, 171.8, 170.9, 169.7, and 168.1 for seven carbonyl carbons, indicating that **3** is a larger peptide than **1** and **2**. The most noticeable difference in the NMR data was the presence of an isolated methyl group suggesting an acetyl group (δ_C_ 23.1, δ_H_ 1.83) (Table 2).

As for the previous compounds, the structure of **3** was determined by combined 2-D NMR data. Consequently, the same five amino acid residues (Ala, *N*-MePhe, Pro, Tyr, and Val) that could be found in **2** were readily identified by the COSY and intra-residual HMBC correlations (Figure 2). Unlike other carbonyl carbons, the carbonyl carbon of Pro failed to show HMBC correlations with neighboring protons and was unassigned at this stage. For the remaining residue, the COSY data revealed a proton spin system linearly consisting of four resonances at δ_H_ 6.29, 4.69, 5.53, and 1.18 for NH-CH-CH-CH_3_, respectively. The proton–proton coupling pattern and remarkably de-shielded methine at δ_H_ 5.53 suggested the presence of a threonine (Thr) residue. This was confirmed by the HMBC data in which the long-range couplings of the methine and methyl protons with neighboring carbons provided not only the linear structure but also the assignment of its carbonyl carbon at δ_C_ 169.7. The previously described acetyl group was also confirmed by a two-bond correlation between the methyl proton and carbonyl carbon at δ_C_ 171.8. Subsequently, the unassigned amide connection of Pro in the cyclopeptide was also confirmed indirectly but logically by constructing an *N*-AcThr residue with the HMBC correlations of NH and its α-methine protons with the carbonyl carbon (Figure 2). Thus, **3** was found to possess this unit in addition to the same five amino acid residues found in **2**. A literature study showed that aspergillicins A–E, cyclohexapeptides from an Australian marine-derived *Aspergillus carneus,* also contained this modified amino-acid residue as a building block [15]. 

The assembly of the amino acid residues into the full structure was accomplished by the interpretation of HMBC data for the intra-residual HMBC correlations. Consequently, as shown in Figure 2, two partial structures, Ala-*N*-MePhe-Tyr and *N*-AcThr-Val, were readily deduced. An ester linkage connected these fragments according to a crucial HMBC correlation between the *β*-CH proton of Thr and the CO carbon of Ala. Although it was not directly indicated by the HMBC data, it was interpreted that the only remaining carbon at δ_C_ 173.3 must be the carbonyl carbon of Pro, which form a peptide bond with the NH of Tyr. 

The structure of **3** derived from its HMBC data was confirmed by high-resolution LC/MS-MS analysis. It was observed that the *N*-AcThr moiety was readily converted to Thr during the analysis. Despite this, as shown in Figure 3, the linear sequence of this compound was independently found to be *N*-MePhe-Ala-*N*-AcThr-Val-Pro-Tyr, securing the placement of Pro between Val and Tyr. Thus, the structure of **3** was defined to be a new cyclohexadepsipeptide. Interestingly, the amino acid sequence of **3** was opposite to those of **2** (also **1**) with reference to the five commonly-presented units. 

The absolute configurations of the amino acids in **3** were assigned by advanced Marfey’s analysis. Unexpectedly, the Val in this compound was found to have a D-configuration by repeated analyses, while all other units had L-configurations (Figure S32, Table S1). In the LC-MS/MS analysis, *N*-AcThr was hydrolyzed to Thr. Additionally, the Marfey’s analysis for the *β*-stereogenic center of Thr assigned this to a common L-Thr. Thus, the structure of **3**, designated to be FJ120DPA, was determined to be a new cyclohexadepsipeptide containing unusual D-Val and L-*N*-AcThr units.

A minor constituent congener of **3**, compound **4,** was isolated as an amorphous solid that was found to have the formula C_38_H_52_N_6_O_10_ by HRFABMS data ([M + Na]^+^ *m/z* 775.3646, calcd for C_38_H_52_N_6_NaO_10,_ 775.3643). Compound **4** was analyzed in MeOH-*d*_4_ because the compound possesses a better solubility in methanol. Furthermore, the NMR data of compound **4** showed a better peak separation as well as minimal conformer formation. Despite compound **4** being analyzed in another solvent, the ^13^C and ^1^H NMR data of this compound were very similar to those of **3** obtained in CDCl_3_ (Table 2). A detailed examination of these data revealed that all the amino acid residues of **3** were exactly the same as those of **4**. These NMR results, in conjunction with the presence of an additional H_2_O unit in its molecular formula, suggested **4** to be a hydrolyzed derivative of **3**, possibly at the ester linkage between the Ala and the *N*-AcThr residue. This interpretation was confirmed by combined 2-D NMR experiments in which the carbon–proton and proton–proton correlations found for these compounds were virtually identical. Thus, compound **4**, designated to be FJ120DPB, was structurally defined to be a hydrolyzed derivative of FJ120DPA (**3**). The structural similarity between **3** and **4** may bring the possibility of abiotic origin of the latter. However, the presence of compound **4** could already be detected in the unprocessed crude extract by LC-ESI-MS analysis. Furthermore, no acid or water was used for the extraction from the semi-solid cultivation medium. Consequently, it was presumed that compound **4** is not an artifact but rather a natural product. 

To investigate the bioactivity of isolated compounds, antimicrobial activities against pathogenic microorganisms and enzyme inhibitory activities toward anti-virulence drug targets SrtA and ICL were first examined in vitro. In bioassays, these compounds were inactive (MIC (minimum inhibitory concentration) > 128 μg/mL) against the Gram-positive and Gram-negative bacterial strains *Enterococcus faecalis* (ATCC19433), *Enterococcus faecium* (ATCC19434), *Staphylococcus aureus* (ATCC25923), *Escherichia coli* (ATCC25922), *Klebsiella pneumoniae* (ATCC10031), and *Salmonella enterica* (ATCC14028). These compounds were also inactive (MIC > 128 μg/mL) against the fungal strains *Aspergillus fumigatus* (HIC6094), *Candida albicans* (ATCC10231), *Trichophyton mentagrophytes* (IFM40996), and *Trichophyton rubrum* (NBRC9185). There have been several investigations for antimicrobial cyclopeptides from natural product screening. A cyclic pentapeptide asperpeptide A isolated from *Aspergillus* sp. XS-20090B15 showed antibacterial activity against *Bacillus cereus* and *Staphylococcus epidermidis* with the same MIC value of 12.5 μM [16]. A cyclic hexapeptide (ASP2397) produced by *Acremonium persicinum* MF-347833 exhibited potent antifungal activity against *A. fumigatus* (MIC = 0.78 μg/mL) [17].

SrtA and ICL are excellent targets for the design and development of new anti-virulence drugs against pathogenic microorganisms. SrtA, a type of transpeptidase, plays a critical role in the pathogenesis of Gram-positive bacteria, including *S. aureus*, by modulating the ability of the bacterium to adhere to host tissue via the covalent anchoring of adhesins and other virulence-associated proteins to cell wall peptidoglycans [18]. The glyoxylate cycle is a sequence of anaplerotic reactions catalyzed by the key enzymes ICL and malate synthase [19]. The expression of *ICL* is upregulated during infection of macrophages by pathogenic microorganisms such as the pulmonary bacterium *Mycobacterium tuberculosis* [20] and the human pathogenic fungus *C. albicans* [21]. The mutant strains lacking *srtA* or *icl* are markedly less virulent in mice than the wild-type without affecting microbial viability [18,20,21]. In further bioassays, these compounds were moderately active against *S. aureus* SrtA and exhibited no antibacterial activity against *S. aureus* growth (MIC > 128 μg/mL). Compound **2** exhibited the strongest inhibitory activity (IC_50_ = 53.1 μM) of the test compounds with the inhibition comparable to the positive controls berberine chloride (IC_50_ = 104.3 μM) and curcumin (IC_50_ = 47.8 μM) (Table 3). In a similar assay, compound **2** showed weak inhibition (IC_50_ = 104.3 μM) of isocitrate lyase (ICL) derived from *C. albicans*. The IC_50_ value of compound **2** is comparable to the previously reported diketopiperazine cyclo(l-Phe-l-Val) (IC_50_ = 109.5 μM) isolated from marine-derived *Streptomyces puniceus* [22].

## 3. Materials and Methods

### 3.1. General Experimental Procedures

Optical rotations were measured on a JASCO P1020 polarimeter (Jasco, Tokyo, Japan) using a 1 cm cell. UV spectra were acquired with a Hitachi U-3010 spectrophotometer (Hitachi High-Technologies, Tokyo, Japan). IR spectra were recorded on a JASCO 4200 FT-IR spectrometer (Jasco, Tokyo, Japan) using a ZnSe cell. ^1^H and ^13^C NMR spectra were measured in DMSO-*d_6_*, CDCl_3_, or CD_3_OD solutions on Bruker Avance –400, –500, –600, or –800 instruments (Billerica, MA, USA). High resolution FAB mass spectrometric data were obtained at the Korea Basic Science Institute (Daegu, Korea) and were acquired using a JEOL JMS 700 mass spectrometer (Jeol, Tokyo, Japan) with *meta*-nitrobenzyl alcohol (NBA) as the matrix. High-resolution LC-MS/MS data were obtained at the National Instrumentation Center for Environmental Management (Seoul, Korea) on a Q-TOF 5600 instrument equipped with a Dionex U-3000 HPLC system. Semi-preparative HPLC separations were performed on a Spectrasystem p2000 equipped with a Spectrasystem RI-150 refractive index detector. All solvents used were spectroscopic grade or distilled from glass prior to use.

### 3.2. Fungal Material

#### 3.2.1. *Aspergillus allahabadii* (Strain Number JG002)

The fungal strain *A. allahabadii* (strain number JG002) was isolated from underwater sediments collected at –20 m off the coast of Jeju-do, Korea, in April 2018. The sample was diluted using sterile seawater. One milliliter of diluted sample was processed utilizing the spread plate method in YPG medium (5 g of yeast extract, 5 g of peptone, 10 g of glucose, 0.15 g of penicillin G, 0.15 g of streptomycin sulfate, 24.8 g of Instant Ocean, and 16 g of agar in 1 L of distilled water) plates. The plates were incubated at 28 °C for 5 days. The strain was identified using standard molecular biology protocols by DNA amplification and sequencing of the ITS region. Genomic DNA extraction was performed using Intron’s i-genomic BYF DNA Extraction Mini Kit according to the manufacturer’s protocol. The strain was identified using standard molecular biology protocols by DNA amplification and sequencing of the ITS region [23]. The 18S rDNA sequence of this strain exhibited 100% identity (564/564) with that of *A. candidus*, *A. allahabadii*, and *A. niveus*. Due to the limitations of the ITS regions to identify intrasection species, sequence comparison of the β-tubulin was further analyzed [24]. This strain exhibited 100% identity with that of *A. allahabadii* strain CGMCC_3.02584 (GenBank accession number MH292842.1) in β-tubulin region (477/477). The nucleotide sequence of JG002 was deposited in the GenBank database under accession number MK424488.

#### 3.2.2. Aspergillus ochraceopetaliformis (Strain Number FJ120)

The fungal strain *A. ochraceopetaliformis* (strain number FJ120) was isolated from underwater sediments collected off the coast of Jeju-do, Korea, in July 2007. The sample was diluted using sterile seawater. The isolation was performed in the same conditions as for the strain JG002. The identification was also processed using the same protocols. The nucleotide sequence of FJ120 was deposited in the GenBank database under accession number KF384187. The 18S rDNA sequence of this strain exhibited 100% identity (588/588) with that of *A. ochraceopetaliformis* strain RKI08-134 (GenBank accession number FJ797698).

### 3.3. Fermentation 

#### 3.3.1. JG002

The fungal strain was cultured on solid YPG media (5 g of yeast extract, 5 g of peptone, 10 g of glucose, 24.8 g of Instant Ocean, and 16 g of agar in 1 L of distilled water) for 7 days. An agar plug (1 × 1 cm) was inoculated in a 250 mL flask containing 100 mL of YPG media. After 7 days of growth, 10 mL of each culture was transferred to 2.8 L Fernbach flasks containing rice media (200 g of rice, 0.5 g of yeast extract, 0.5 g of peptone, and 12.4 g of Instant Ocean in 200 mL of distilled water). In total, 800 g of rice media was prepared and cultivated for 4 weeks at 28 °C, with agitating once every week.

#### 3.3.2. FJ120

The seed preparation and inoculation were performed under the same culturing conditions as for the strain JG002. Then, 10 mL of each culture was transferred to 2.8 L Fernbach flasks containing YMM media (5g of yeast extract, 5g of malt extract, 10g of mannitol and 24.8 g of Instant Ocean in 1000 mL of distilled water). In total, 20 L of YMM media was prepared and cultivated under static conditions for 8 weeks at 28 °C.

### 3.4. Extraction and Isolation

#### 3.4.1. JG002

The entire culture was macerated and extracted with MeOH (1 L × 3). The solvent was evaporated in vacuo to afford a brown organic gum (4.2 g). The extract was separated by C_18_ reversed-phase vacuum flash chromatography using sequential mixtures of H_2_O and MeOH (six fractions of H_2_O-MeOH, gradient from 50:50 to 0:100), acetone, and finally EtOAc as the eluents. Based on the results of ^1^H NMR analysis, the fractions eluted with H_2_O-MeOH 40:60 (380 mg) and 30:70 (760 mg) were chosen for further separation. The fraction that eluted with H_2_O-MeOH (40:60) was separated by semi-preparative reversed-phase HPLC (YMC-ODS-A column, 250 × 10 mm, 5 μm; H_2_O-MeCN, 70:30, 2.0 mL/min), affording compound **1**. The H_2_O-MeOH (30:70) fraction from vacuum flash chromatography was separated by semi-preparative reversed-phase HPLC (H_2_O-MeCN, 58:42, 2.0 mL/min), and afforded compound **2**. The overall isolated amounts were 10.3 and 5.8 mg for **1** and **2**, respectively.

#### 3.4.2. FJ120

The entire culture was filtered and extracted with EtOAc (20 L × 3). The solvent was evaporated in vacuo to afford a brown organic gum (5.2 g). The extract was separated by C_18_ reversed-phase vacuum flash chromatography using sequential mixtures of H_2_O and MeOH (five fractions of H_2_O-MeOH, gradient from 80:20 to 0:100), finally MeOH:MC (1:1) as the eluents. Based on the results of ^1^H NMR analysis, the fractions eluted with H_2_O-MeOH 80:20 (1370 mg) and 40:60 (1380 mg) were chosen for further separation. The fraction that was eluted with H_2_O-MeOH (80:20) was separated by semi-preparative reversed-phase HPLC (YMC-ODS-A column, 250 x 10 mm, 5 μm; H_2_O-MeOH, 50:50, 2.0 mL/min), affording compound **4**. This compound was further purified by reversed-phase HPLC (YMC-ODS-A column, 4.6 × 250 nm, 5 μm; H_2_O-MeCN, 85:15, 0.7 mL/min). The H_2_O-MeOH (40:60) fraction from vacuum flash chromatography was separated by semi-preparative reversed-phase HPLC (H_2_O-MeOH, 50:50, 2.0 mL/min) and afforded pure compound **3**. The overall isolated amounts were 24.6 and 3.8 mg for **3** and **4**, respectively.

JG002CPA (**1**): Pale yellow, amorphous solid, [α]${}_{D}^{25}$ +61 (*c* 0.20, MeOH); UV (MeOH) *λ*_max_ (log ε) 218 (3.48), 277 (2.68) nm; ^1^H and ^13^C NMR: see Table 1; IR (ZnSe) *ν*_max_ 3326, 2956, 1649, 1517, 1425 cm^−1^; HRFABMS, *m/z* 606.3296 [M + H]^+^ (calcd for C_33_H_44_N_5_O_6,_ 606.3292). 

JG002CPB (**2**): Pale yellow, amorphous solid, [α]${}_{D}^{25}$ +69 (*c* 0.20, MeOH); UV (MeOH) *λ*_max_ (log ε) 221 (3.47), 281 (2.67) nm; ^1^H and ^13^C NMR: see Table 1; IR (ZnSe) *ν*_max_ 3300, 2971, 2920, 1649, 1512, 1452 cm^−1^; HRFABMS, *m/z* 592.3133 [M + H]^+^ (calcd for C_32_H_42_N_5_O_6,_ 592.3135).

FJ120DPA (**3**): Pale yellow, amorphous solid, [α]${}_{D}^{25}$ +66 (*c* 0.20, MeOH); UV (MeOH) *λ*_max_ (log ε) 212 (3.56), 280 (2.16) nm; ^1^H and ^13^C NMR: see Table 2; IR (ZnSe) *ν*_max_ 3304, 2964, 2922, 1732, 1644, 1540, 1514, 1453 cm^−1^; HRFABMS, *m/z* 735.3721 [M + H]^+^ (calcd for C_38_H_51_N_6_O_9,_ 735.3718).

FJ120DPB (**4**): Pale yellow, amorphous solid, [α]${}_{D}^{25}$ +73 (*c* 0.20, MeOH); UV (MeOH) *λ*_max_ (log ε) 220 (3.57), 280 (2.65) nm; ^1^H and ^13^C NMR: see Table 2; IR (ZnSe) *ν*_max_ 3315, 2953, 2912, 1685, 1649, 1546, 1453 cm^−1^; HRFABMS, *m/z* 775.3646 [M + Na]^+^ (calcd for C_38_H_52_N_6_NaO_10,_ 775.3643).

### 3.5. Stereochemical Analysis of the Amino Acid Residues

Compounds **1**–**3** (1 mg each) were hydrolyzed in 0.5 mL of 6 N HCl at 120 °C for 8 h. After removing the HCl in vacuo, 0.5 mg portions of the hydrolysate were transferred into two vials and dissolved in 100 μL of 1 N NaHCO_3_. L-FDAA (100 μL of 10 mg/mL in acetone) was added to one reaction vial, and D-FDAA was added to the other vial. The reactions were maintained at 80 °C for 3 min. Then, the reaction mixtures were neutralized by adding 50 μL of 2 N HCl and diluted with 300 μL of 50% aqueous CH_3_CN−H_2_O. Twenty microliters of each reaction mixture were analyzed by LC/MS using a Phenomenex C_18_(2) column (Luna, 100 × 4.6 mm, 5 μm) with gradient solvent conditions (flow rate 0.7 mL/min; UV 340 nm detection; 10% to 60% CH_3_CN−H_2_O with 0.1% formic acid over 50 min). L-FDAA derivatives were eluted before D-FDAA derivatives for all amino acid residues in the hydrolysate of **1** and **2**. Thus, the absolute configurations of all the amino acid residues in these compounds were determined to be L. D-FDAA derivative eluted before L-FDAA derivative for Val residue in the hydrolysate of **3**, while other amino acid residues eluted the L-FDAA derivatives faster than the D-FDAA derivatives (Supporting Information). In order to assign between L-Thr and L-*allo*-Thr in **3**, a Marfey’s analysis using additional HPLC analysis with a YMC-ODS-A column (250 × 4.6 mm, 5 μm) and gradient solvent conditions (flow rate 0.7 mL/min; UV 360 nm detection; 20% to 90% MeOH−H_2_O with 0.1% TFA over 60 min) was applied, and the residue was determined to be L-Thr rather than L-*allo*-Thr.

### 3.6. Biological Assays

#### 3.6.1. Antibacterial Activity Assay

The antibacterial activity assay was performed according to the Clinical and Laboratory Standards Institute (CLSI) method [25]. Gram-positive bacteria (*S. aureus* ATCC25923, *E. faecalis* ATCC19433, *E. faecium* ATCC19434) and Gram-negative bacteria (*K. pneumoniae* ATCC10031, *S. enterica* ATCC14028, *E. coli* ATCC25922) were cultured in MHB (Mueller Hinton broth) at 37 °C. Each test compound was dissolved in dimethyl sulfoxide (DMSO) and diluted with MBH to prepare serial twofold dilutions in the range of 0.06–128 μg/mL (final DMSO concentration: 1%). An aliquot of 190 μL of MBH containing the test compound was mixed with 10 μL of the broth containing approximately 10^6^ colony-forming units (cfu)/mL of test bacterium (final concentration: 5 × 10^4^ cfu/mL) in each well of a 96-well plate. The plates were incubated for 24 h at 37 °C. The MIC was defined as the lowest concentration of test compound that prevented cell growth. Ampicillin and tetracycline were used as reference compounds. 

#### 3.6.2. Antifungal Activity Assay

The antifungal activity assay was performed in accordance with the guidelines in CLSI document M38 [26]. *C. albicans* ATCC10231, *A. fumigatus* HIC6094, *T. rubrum* NBRC9185, and *T. mentagrophytes* IFM40996 were used as test organisms. Each stock solution of the compound was diluted in RPMI 1640 broth with the concentration range of 0.06–128 μg/mL. The final inoculum concentration was 10^4^ cells/mL in each well of a 96-well plate. The MIC was defined after incubation for 24 h (for *C. albicans*), 48 h (for *A. fumigatus*), and 96 h (for *T. rubrum* and *T. mentagrophytes*) at 37 °C. Amphotericin B was used as a positive control.

#### 3.6.3. ICL Inhibition Assay

The recombinant ICL from *C. albicans* was prepared and the inhibitory activity of the test compounds against ICL was determined by previously documented procedures [23]. The reaction mixture consisted of 20 mM sodium phosphate buffer (pH 7.0), 4.1 mM phenylhydrazine, 3.75 mM MgCl_2_, 1.27 mM threo-dl(+)isocitrate, and 2.5 μg/mL ICL, and was incubated with the test compounds at a concentration range of 1 to 128 μg/mL at 37 °C for 30 min. The increase in intensity of absorbance resulting from the formation of glyoxylate phenylhydrazone was observed using an UVmini-1240 spectrophotometer (SHIMADZU, Tokyo, Japan) at a wavelength of 324 nm. A known ICL inhibitor, 3-nitropropionic acid, was used as a reference control.

#### 3.6.4. SrtA Inhibition Assay

*S*. *aureus*-derived recombinant SrtA was purified from transformed *E. coli* by nickel-based affinity chromatography, and enzyme activity was determined by quantifying the intensity of augmented fluorescence upon cleavage of a synthetic peptide containing LPETG motifs [27]. For the reactions, 300 μL of reaction mixture (50 mM Tris–HCl, pH 7.5, 150 mM NaCl, 5 mM CaCl_2_, 55 μg SrtA, and 0.75 μg synthetic peptide substrate, Dabcyl-QALPETGEE-Edans) was added to a 96-well microtiter plate. After incubation at 37 °C for 1 h, the increase in the fluorescence intensity was recorded by an UVmini-1240 spectrophotometer (SHIMADZU, Tokyo, Japan) using excitation and emission wavelengths of 350 and 495 nm, respectively. Curcumin and berberine chloride were used as positive controls.

## 4. Conclusions

Four new peptides were isolated and structurally elucidated from the culture broths of marine-derived fungi *A. allahabadii* and *A. ochraceopetaliformis.* Based upon the results of combined chemical and spectroscopic analyses, the amino acid sequences of two cyclopentapeptides (**1** and **2**) from *A. allahabadii* were elucidated. Furthermore, one cyclodepsihexapeptide (**3**) and its linear analog (**4**) from *A. ochraceopetaliformis* could be elucidated. In addition, the presence of a D-Val residue in the two hexapeptides was also found by Marfey’s analysis. The new compounds moderately inhibited the microbial enzyme sortase A and (**2**) weakly inhibited isocitrate lyase.

## Figures and Tables

**Table 1:** NMR data of compounds **1** and **2** in DMSO-*d_6_*.

		1		2	
Unit	Position	δ_C,_ Type	δ_H,_ Mult (*J* in Hz)	δ_C,_ Type	δ_H,_ Mult (*J* in Hz)
Ala	CO	171,2, C		171.2, C	
	α	44.2, CH	3.54, dd (7.9, 6.8)	43.9, CH	3.60, dd (8.0, 6.8)
	*β*	17.1, CH_3_	0.70, d (6.4)	17.1 CH_3_	0.71, d (6.4)
	NH		8.29, d (8.4)		8.32, d (8.3)
*N*-Me-Phe	CO	168.1, C		168.2, C	
	α	61.7, CH	4.17, dd (11.6, 3.6)	61.6, CH	4.16, dd (11.4, 3.4)
	*β*	33.9, CH_2_	3.24, dd (14.3, 3.3)	33.9, CH_2_	3.25, dd (14.3, 3.3)
			2.70, dd (14.4, 11.8)		2.72, dd (14.4, 11.6)
	*γ*	137.5, C		137.5, C	
	*ortho*	129.0, CH	7.09, d (7.2)	129.0, CH	7.10, d (7.2)
	*meta*	128.5, CH	7.28, d (7.6)	128.5, CH	7.28, d (7.6)
	*para*	126.7, CH	7.22, t (7.4)	126.7, CH	7.22, t (7.4)
	N-CH_3_	30.5, CH_3_	2.61, s	30.5, CH_3_	2.63, s
Tyr	CO	168.9, C		168.9, C	
	α	52.6, CH	4.78, td (8.5, 5.2)	52.7, CH	4.76, td (8.5, 5.2)
	*β*	37.3, CH_2_	3.04, dd (13.4, 8.8)	37.3, CH_2_	3.02, dd (13.4, 9.0)
			2.73, dd (13.5, 5.7)		2.74, dd (13.5, 4.7)
	*γ*	127.4, C		127.4, C	
	*ortho*	130.2, CH	7.04, d (8.4)	130.2, CH	7.04, d (8.4)
	*meta*	114.9, CH	6.66, d (8.4)	114.9, CH	6.66, d (8.4)
	*para*	155.7, C		155.8, C	
	OH		9.19, s		9.21, s
	NH		7.27, m		7.26, m
Pro	CO	170.6, C		170.6, C	
	α	60.9, CH	4.10, dd (7.9, 1.7)	60.9, CH	4.10, dd (7.3, 2.6)
	*β*	31.5, CH_2_	1.95, m	31.5, CH_2_	1.93, m
			1.88, m		
	*γ*	21.4, CH_2_	1.77, m	21.5, CH_2_	1.78, m
			1.58, m		1.61, dq (12.3, 9.1)
	*δ*	46.4, CH_2_	3.47, m	46.2, CH_2_	3.49, m
			3.37, m		3.35, m
Leu	CO	170.4, C			
	α	53.3, CH	4.19, m		
	*β*	41.3, CH_2_	1.37, m		
			1.34, m		
	*γ*	24.5, CH	1.37, m		
	*δ*	22.4, CH_3_	0.88, d (6.1)		
		21.7, CH_3_	0.80, d (6.2)		
	NH		6.96, d (9.0)		
Val	CO			169.2, C	
	α			61.0, CH	3.82, t (9.6)
	*β*			30.7, CH	1.69, m
	*γ*			19.3, CH_3_	0.80, d (6.6)
				18.9, CH_3_	0.78, d (6.7)
	NH				7.00, d (8.7)

**Table 2:** NMR data of compounds **3** and **4**.

		3 *^a^*		4 *^b^*	
Unit	Position	δ_C,_ Type	δ_H,_ Mult (*J* in Hz)	δ_C,_ Type	δ_H,_ Mult (*J* in Hz)
Val	CO	172.0, C		172.8, C	
	α	57.1, CH	4.43, t (9.8)	59.4, CH	4.10, d (9.0)
	*β*	30.3, CH	1.89, m	30.8, CH	1.97, m
	*γ*	19.2, CH_3_	0.86, d (6.5)	19.4, CH_3_	1.02, d (6.6)
		18.8, CH_3_	0.92, d (6.5)	19.2, CH_3_	0.91, d (6.8)
	NH		6.43, d (9.1)		
Pro	CO	173.3, C		173.7, C	
	α	59.5, CH	4.31, d (5.8)	61.7, CH	4.35, dd (8.3, 2.4)
	*β*	29.7, CH_2_	2.13, m	30.5, CH_2_	1.88, m
					1.74, m
	*γ*	24.7, CH_2_	1.88, m	24.2, CH_2_	1.63, m
					1.04, m
	*δ*	48.1, CH_2_	3.93, m	48.6, CH_2_	3.72, m
			3.52, m		3.46, m
Tyr	CO	172.4, C		173.9, C	
	α	50.6, CH	4.54, dd (13.3, 6.6)	51.2, CH	4.62, dd (11.2, 4.0)
	*β*	35.9, CH_2_	2.42, dd (13.6, 9.2)	36.0, CH_2_	2.63, dd (13.1, 11.5)
			1.87, dd (13.2, 6.2)		1.43, dd (13.2, 3.5)
	*γ*	126.6, C		129.2, C	
	*ortho*	130.2, CH	6.77, d (7.8)	131.5, CH	6.92, d (8.3)
	*meta*	115.9, CH	6.66, d (8.0)	115.9, CH	6.64, d (8.4)
	*para*	155.9, C		157.3, C	
	NH		6.44, d (9.1)		
*N*-Me-Phe	CO	168.1, C		170.5, C	
	α	63.2, CH	4.82, t (6.8)	63.7, CH	5.28, dd (10.9, 3.4)
	*β*	34.5, CH_2_	3.34, dd (14.0, 5.7)	35.4, CH_2_	3.16, dd (14.3, 3.5)
			2.52, dd (13.9, 7.8)		2.97, dd (14.1, 9.3)
	*γ*	137.9, C		139.4, C	
	*ortho*	129.4, CH	7.13, d (7.4)	130.7, CH	7.28, d (7.2)
	*meta*	129.1, CH	7.24, d (7.5)	130.1, CH	7.30, d (7.3)
	*para*	127.3, CH	7.23, d (7.3)	128.1, CH	7.17, d (7.5)
	N-CH_3_	29.5, CH_3_	2.87, s	30.2, CH_3_	2.90, s
Ala	CO	170.9, C		180.8, C	
	α	48.5, CH	4.67, d (7.9)	52.1, CH	4.19, m
	*β*	18.6, CH_3_	1.32, d (7.3)	18.5, CH_3_	1.34, d (6.5)
	NH		8.16, d (8.3)		
*N*-Ac-Thr	CO	169.7, C		173.0, C	
	α	55.4, CH	4.69, m	59.7, CH	4.47, d (5.2)
	*β*	70.1, CH	5.53, q (5.9)	68.6, CH	4.17, q (5.9)
	*γ*	17.0, CH_3_	1.18, d (6.5)	19.7, CH_3_	1.21, d (6.4)
	NH		6.29, d (9.0)		
	CO	171.8, C		174.1, C	
	α	23.1, CH_3_	1.83, s	22.8, CH_3_	2.09, s

*^a,b^* Measured in CDCl_3_ and CD_3_OD, respectively.

**Table 3:** Results of bioactivity tests.

	IC_50_ (μM)	
Compound	SrtA Inhibition	ICL Inhibition
**1**	70.0	>128
**2**	53.1	116.8
**3**	131.9	>128
**4**	77.0	>128
Berberine chloride ^a^	104.3	
Curcumin ^a^	47.8	
3-Nitropropionic acid ^a^		18.5

*^a^* Positive control.

## Supplementary Materials

The following are available online at https://www.mdpi.com/1660-3397/17/9/488/s1. Figures S1–S28: HRFABMS, 1-D and 2-D NMR spectra of **1**–**4**, Figures S29–S31: HRLC/MS-MS fragmentation analysis of **1**–**3**, Figure S32: The advanced Marfey’s analysis of **1**–**4**, Table S1: The advanced Marfey’s analysis of **1**–**4**, Figure S33: The LC analysis of L-FDAA derivatives of Thr and *allo*-Thr for **3**, Figure S34: The LC-MS analysis of FJ120 extract, Tables S2-S5: The NMR table of compound **1**–**4**.
