# Supplementary material for: New Peptides from The Marine-Derived Fungi Aspergillus allahabadii and Aspergillus ochraceopetaliformis
Source: Mar Drugs. 2019 Aug 21;17(9):488. doi: 10.3390/md17090488 (PMC6780696; doi:10.3390/md17090488)
Supplement: Supplementary file 1 [file marinedrugs-17-00488-s001.pdf]

Supporting Information

New Peptides from the Marine-Derived Fungi *Aspergillus allahabadii* and *Aspergillus ochraceopetaliformis*

Ji-Yeon Hwang,[1] Jung-Ho Lee,[1] Sung Chul Park,[1] Jayho Lee,[2] Dong-Chan Oh,[1] Ki-Bong Oh,[2,*] and Jongheon Shin[1,*]

[1]*Natural Products Research Institute, College of Pharmacy, Seoul National University, San 56-1, Sillim, Gwanak, Seoul 151-742, Korea*

[2]*Department of Agricultural Biotechnology, College of Agricultural and Life Science, Seoul National University, San 56-1, Sillim, Gwanak, Seoul 151-921, Korea*

List of Supporting Information

Figure S1	The ^{1}H NMR spectrum of JG002CPA (**1**) (600MHz, DMSO-d_6)	S3
Figure S2	The ^{13}C NMR spectrum of JG002CPA (**1**) (100MHz, DMSO-d_6)	S4
Figure S3	The HSQC spectrum of JG002CPA (**1**) (600MHz, DMSO-d_6)	S5
Figure S4	The COSY spectrum of JG002CPA (**1**) (600MHz, DMSO-d_6)	S6
Figure S5	The HMBC spectrum of JG002CPA (**1**) (600MHz, DMSO-d_6)	S7
Figure S6	The TOCSY spectrum of JG002CPA (**1**) (600MHz, DMSO-d_6)	S8
Figure S7	The HRFABMS data of JG002CPA (**1**)	S9
Figure S8	The ^{1}H NMR spectrum of JG002CPB (**2**) (800MHz, DMSO-d_6)	S10
Figure S9	The ^{13}C NMR spectrum of JG002CPB (**2**) (200MHz, DMSO-d_6)	S11
Figure S10	The HSQC spectrum of JG002CPB (**2**) (800MHz, DMSO-d_6)	S12
Figure S11	The COSY spectrum of JG002CPB (**2**) (800MHz, DMSO-d_6)	S13
Figure S12	The HMBC spectrum of JG002CPB (**2**) (800MHz, DMSO-d_6)	S14
Figure S13	The TOCSY spectrum of JG002CPB (**2**) (600MHz, DMSO-d_6)	S15
Figure S14	The HRFABMS data of JG002CPB (**2**)	S16
Figure S15	The ^{1}H NMR spectrum of FJ120DPA (**3**) (500MHz, $CDCl_3$)	S17
Figure S16	The ^{13}C NMR spectrum of FJ120DPA (**3**) (125MHz, $CDCl_3$)	S18
Figure S17	The HSQC spectrum of FJ120DPA (**3**) (500MHz, $CDCl_3$)	S19
Figure S18	The COSY spectrum of FJ120DPA (**3**) (500MHz, $CDCl_3$)	S20
Figure S19	The HMBC spectrum of FJ120DPA (**3**) (500MHz, $CDCl_3$)	S21
Figure S20	The TOCSY spectrum of FJ120DPA (**3**) (500MHz, $CDCl_3$)	S22
Figure S21	The HRFABMS data of FJ120DPA (**3**)	S23
Figure S22	The ^{1}H NMR spectrum of FJ120DPB (**4**) (800MHz, CD_3OD)	S24
Figure S23	The ^{13}C NMR spectrum of FJ120DPB (**4**) (200MHz, CD_3OD)	S25
Figure S24	The HSQC spectrum of FJ120DPB (**4**) (500MHz, CD_3OD)	S26
Figure S25	The COSY spectrum of FJ120DPB (**4**) (600MHz, CD_3OD)	S27
Figure S26	The HMBC spectrum of FJ120DPB (**4**) (800MHz, CD_3OD)	S28
Figure S27	The TOCSY spectrum of FJ120DPB (**4**) (600MHz, CD_3OD)	S29
Figure S28	The HRFABMS data of FJ120DPB (**4**)	S30
Figure S29	The high-resolution LC/MS-MS fragmentation analysis of JG002CPA (**1**)	S31
Figure S30	The high-resolution LC/MS-MS fragmentation analysis of JG002CPB (**2**)	S32
Figure S31	The high-resolution LC/MS-MS fragmentation analysis of FJ120DPA (**3**)	S33
Figure S32	The LC analysis of L- and D-FDAA derivatives of the amino acid-derived units in compounds **1-4**	S37
Table S1	The LC/MS analysis of L- and D-FDAA derivatives of the amino acid-derived units in compounds **1-4**	S38
Figure S33	The LC analysis of L-FDAA derivatives of Thr and *allo*-Thr for FJ120DPA (**3**)	S39
Figure S34	The LC-MS analysis of FJ120	S40
Table S2	The NMR data of JG002CPA (**1**) in DMSO-d_6	S41
Table S3	The NMR data of JG002CPB (**2**) in DMSO-d_6	S42
Table S4	The NMR data of FJ120DPA (**3**) in $CDCl_3$	S43
Table S5	The NMR data of FJ120DPB (**4**) in CD_3OD	S44

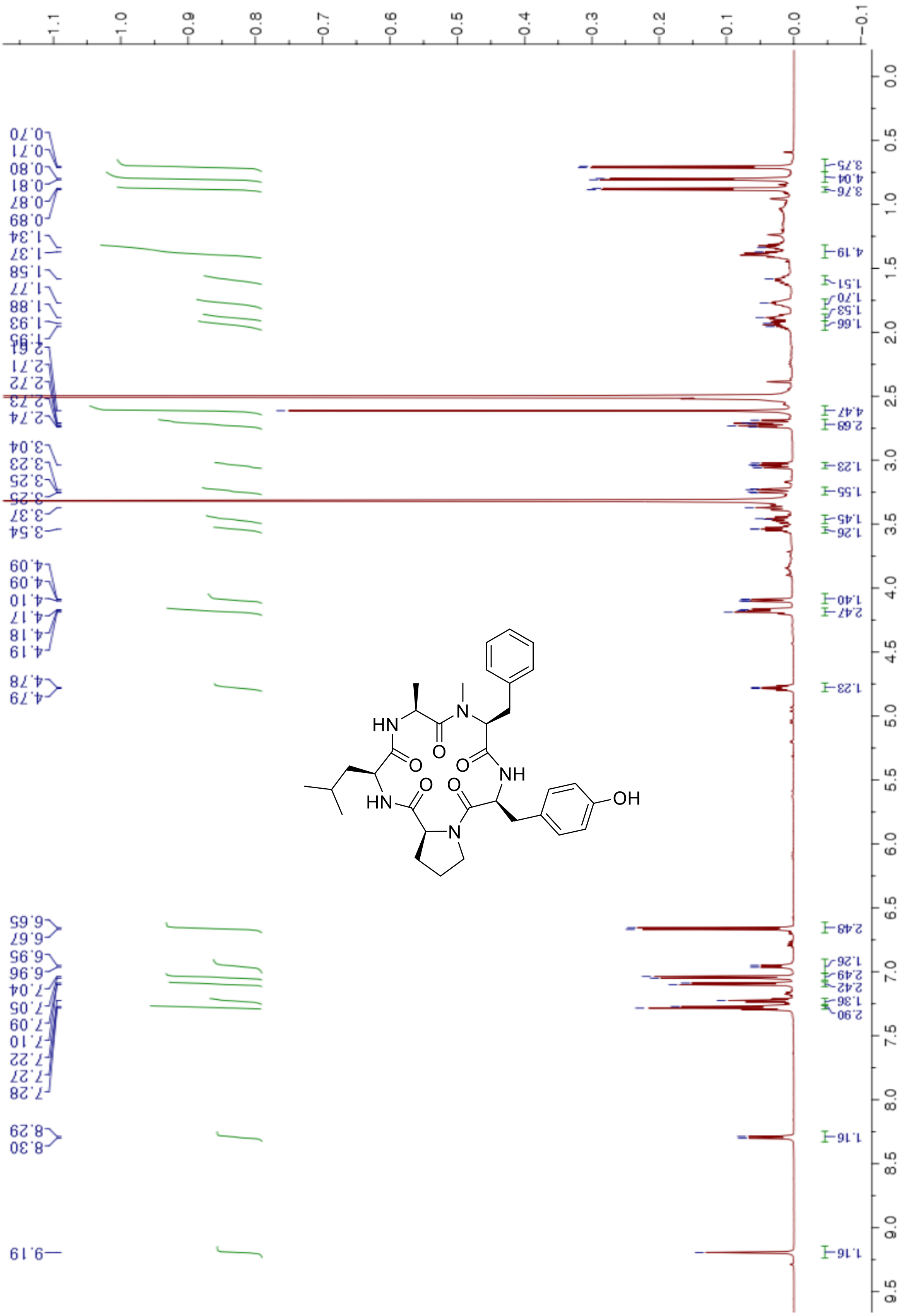

Figure S1. The ^{1}H NMR spectrum of JG002CPA (**1**) (600MHz, DMSO-*d*$_6$)

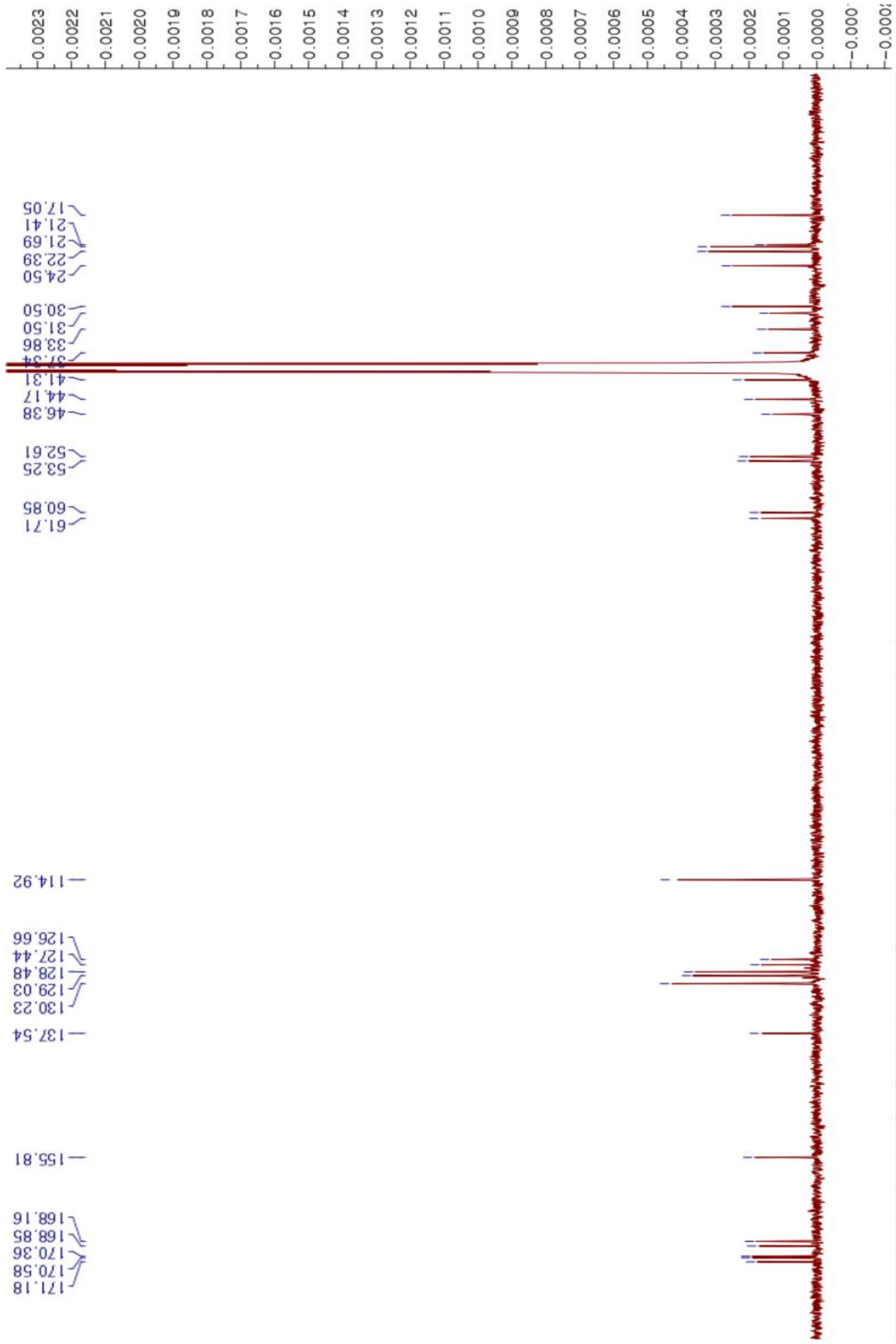

Figure S2. The ^{13}C NMR spectrum of JG002CPA (**1**) (100MHz, DMSO-d_6)

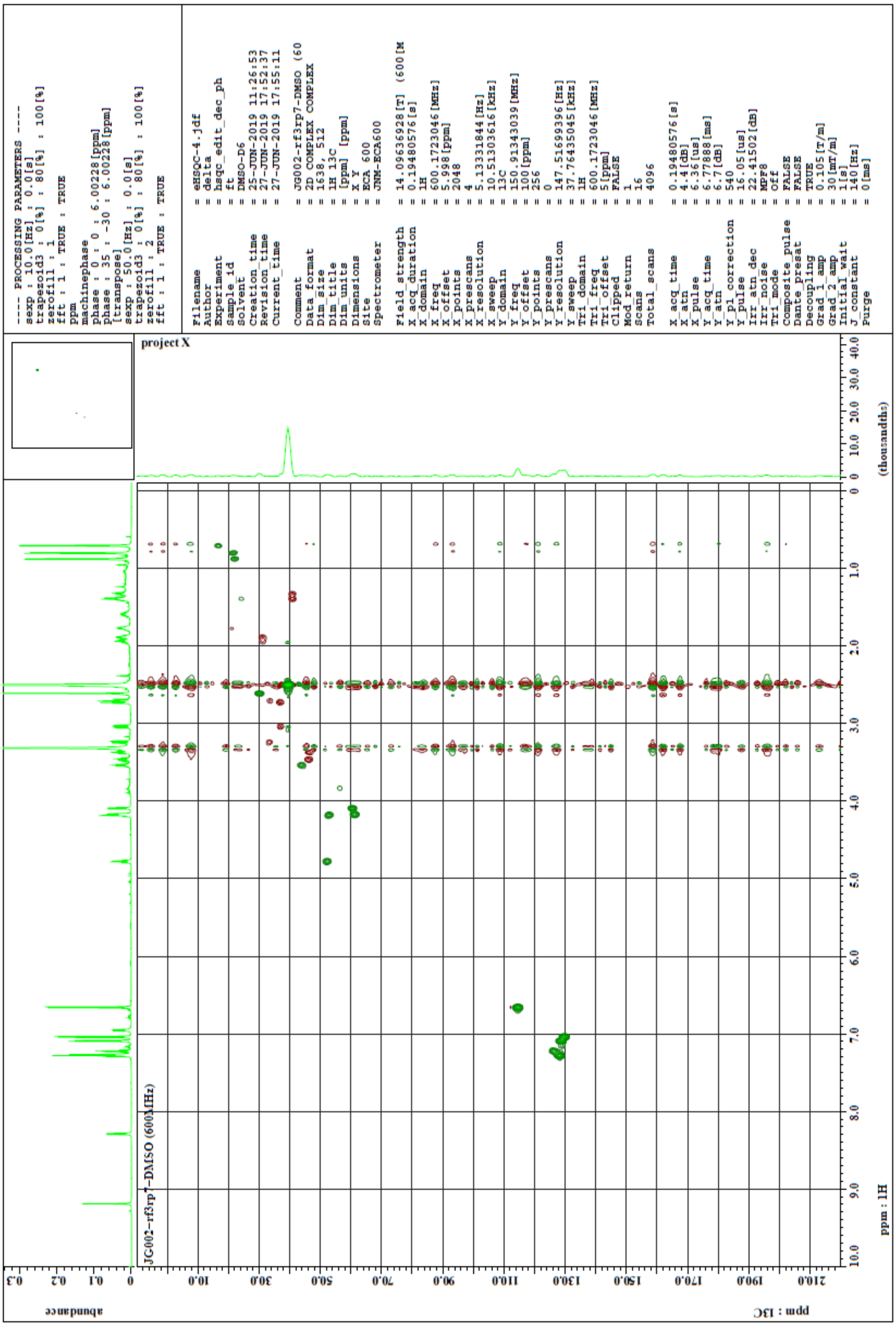

Figure S3. The HSQC spectrum of JG002CPA (**1**) (600MHz, DMSO-d_6)

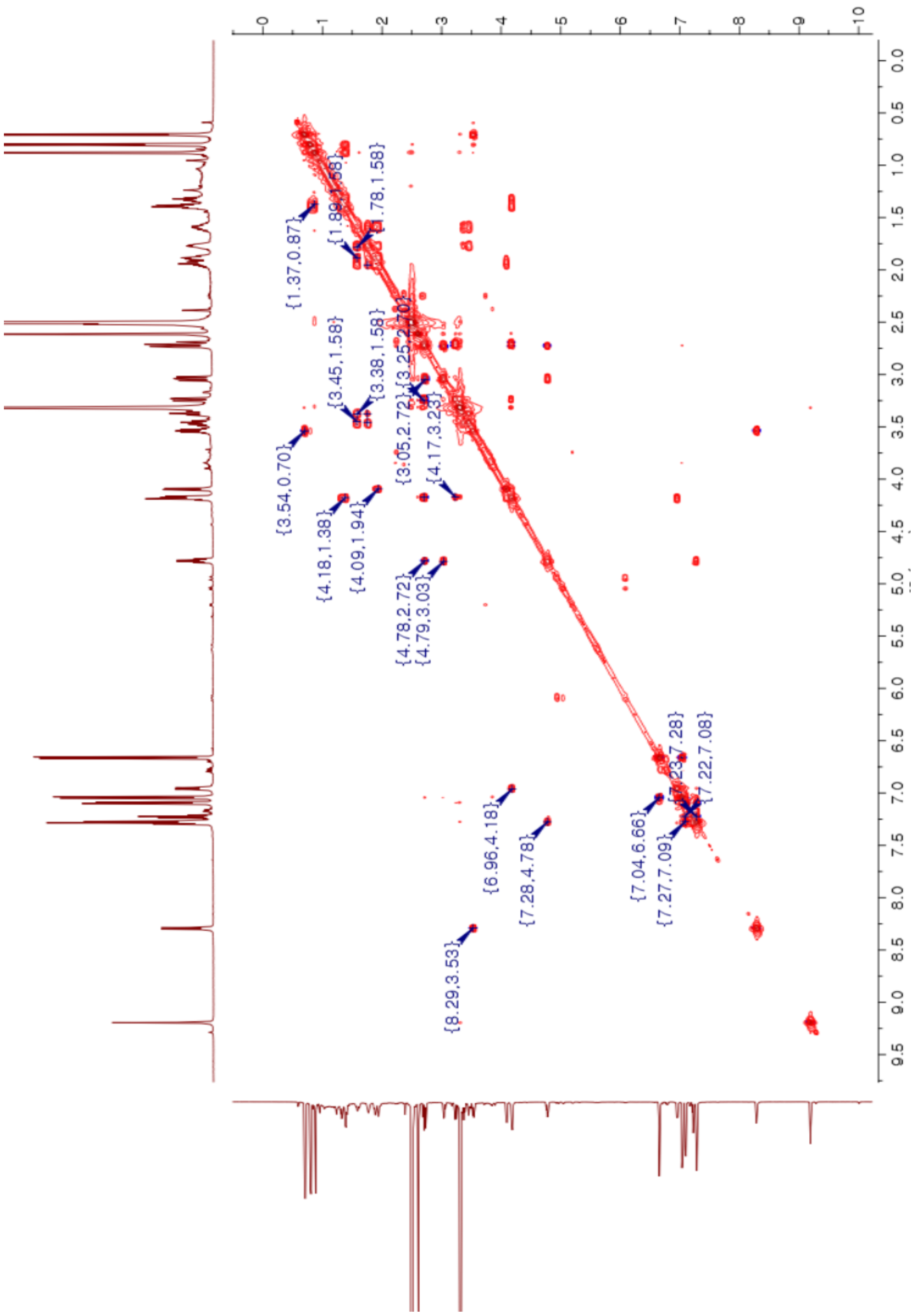

Figure S4. The COSY spectrum of JG002CPA (**1**) (600MHz, DMSO-d_6)

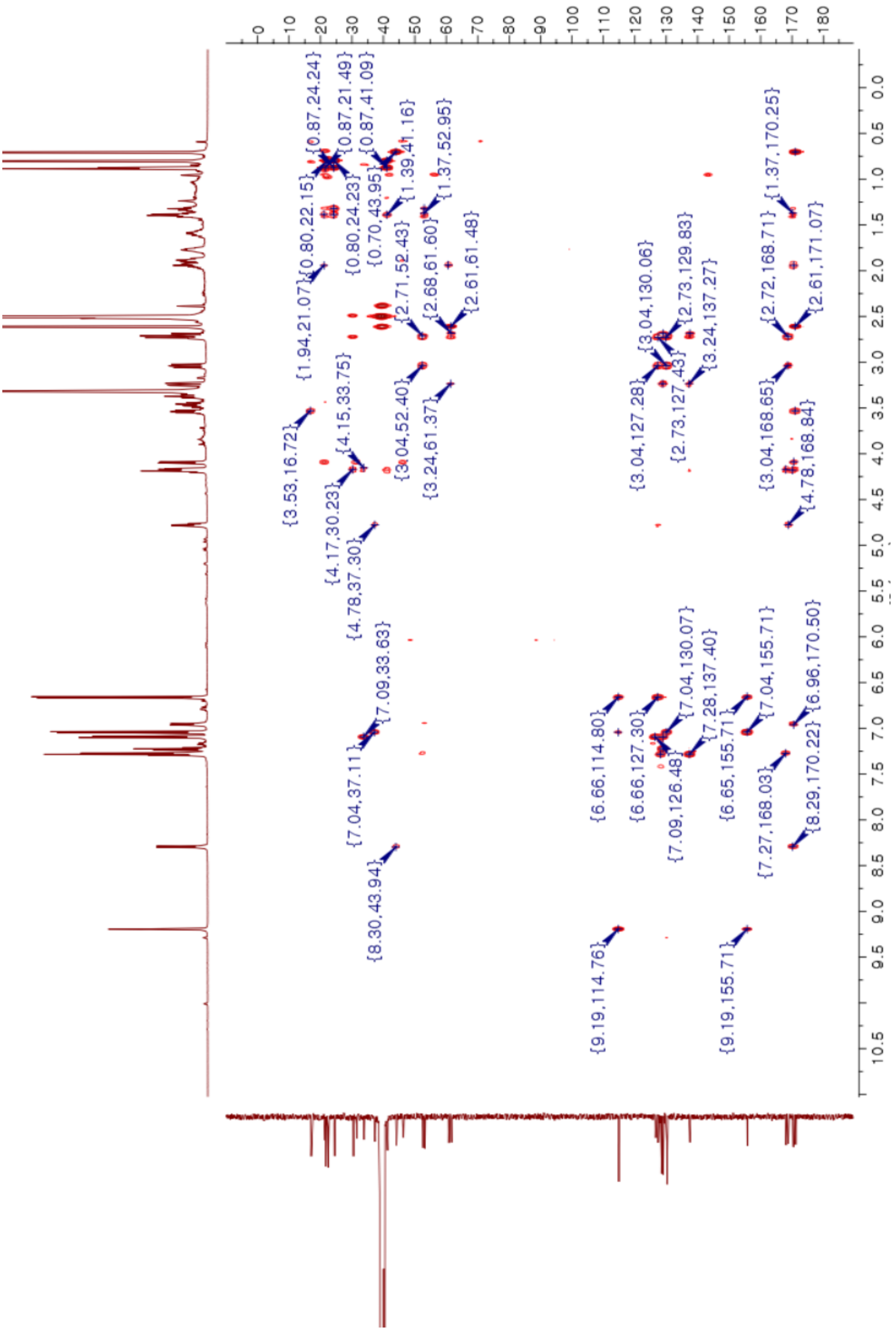

Figure S5. The HMBC spectrum of JG002CPA (**1**) (600MHz, DMSO-d_6)

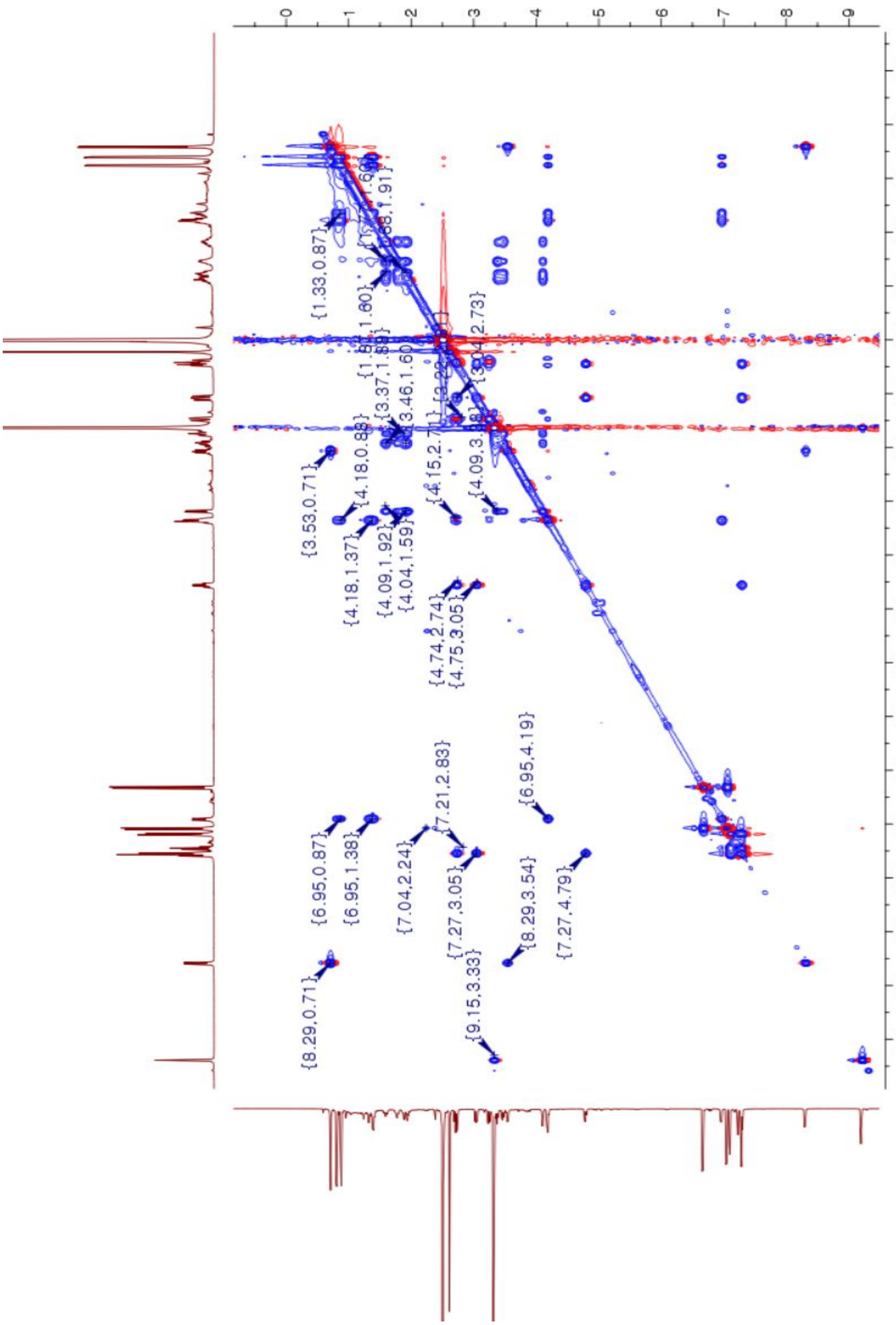

Figure S6. The TOCSY spectrum of JG002CPA (**1**) (600MHz, DMSO-d_6)

[Elemental Composition]
Data : FAB-S655 Date : 07-Jun-2019 14:51
Sample: 1[JG002-RF3RP7]
Note : m-NBA
Inlet : Direct Ion Mode : FAB+
RT : 0.19 min Scan#: (6,11)
Elements : C 100/0, H 100/0, N 10/0, O 10/0
Mass Tolerance : 20ppm, 5mmu if m/z < 250, 10mmu if m/z > 500
Unsaturation (U.S.) : -0.5 - 50.0

Observed m/z	Int%	Err[ppm / mmu]	U.S.	Composition
606.3296	18.7	+1.5 / +0.9	27.0	C 47 H 42
		-8.1 / -4.9	23.5	C 39 H 40 N 7
		+12.6 / +7.6	24.0	C 38 H 38 N 8
		-10.3 / -6.3	23.0	C 41 H 42 N 4 O
		+10.4 / +6.3	23.5	C 40 H 40 N 5 O
		-12.6 / -7.6	22.5	C 43 H 44 N O 2
		+8.2 / +5.0	23.0	C 42 H 42 N 2 O 2
		-1.5 / -0.9	19.5	C 34 H 40 N 9 O 2
		-3.7 / -2.2	19.0	C 36 H 42 N 6 O 3
		-5.9 / -3.6	18.5	C 38 H 44 N 3 O 4
		+14.8 / +9.0	19.0	C 37 H 42 N 4 O 4
		-15.6 / -9.5	15.0	C 30 H 42 N 10 O 4
		-8.1 / -4.9	18.0	C 40 H 46 O 5
		+12.6 / +7.6	18.5	C 39 H 44 N O 5
		+2.9 / +1.8	15.0	C 31 H 42 N 8 O 5
		+0.7 / +0.4	14.5	C 33 H 44 N 5 O 6
		-1.5 / -0.9	14.0	C 35 H 46 N 2 O 7
		-11.2 / -6.8	10.5	C 27 H 44 N 9 O 7
		+9.6 / +5.8	11.0	C 26 H 42 N 10 O 7
		-13.4 / -8.1	10.0	C 29 H 46 N 6 O 8
		+7.3 / +4.5	10.5	C 28 H 44 N 7 O 8
		-15.6 / -9.5	9.5	C 31 H 48 N 3 O 9
		+5.1 / +3.1	10.0	C 30 H 46 N 4 O 9
		+2.9 / +1.8	9.5	C 32 H 48 N O 10
		-6.8 / -4.1	6.0	C 24 H 46 N 8 O 10
		+14.0 / +8.5	6.5	C 23 H 44 N 9 O 10

[Theoretical Ion Distribution] Page: 1
Molecular Formula : C33 H44 N5 O6
(m/z 606.3292, MW 606.7423, U.S. 14.5)
Base Peak : 606.3292, Averaged MW : 606.7380(a), 606.7387(w)

m/z	INT.	
606.3292	100.0000	**
607.3322	38.7690	**********************
608.3350	8.5105	*****
609.3377	1.3581	*
610.3403	0.1731	
611.3428	0.0185	
612.3454	0.0017	
613.3479	0.0001	

Figure S7. The HRFABMS data of JG002CPA (**1**)

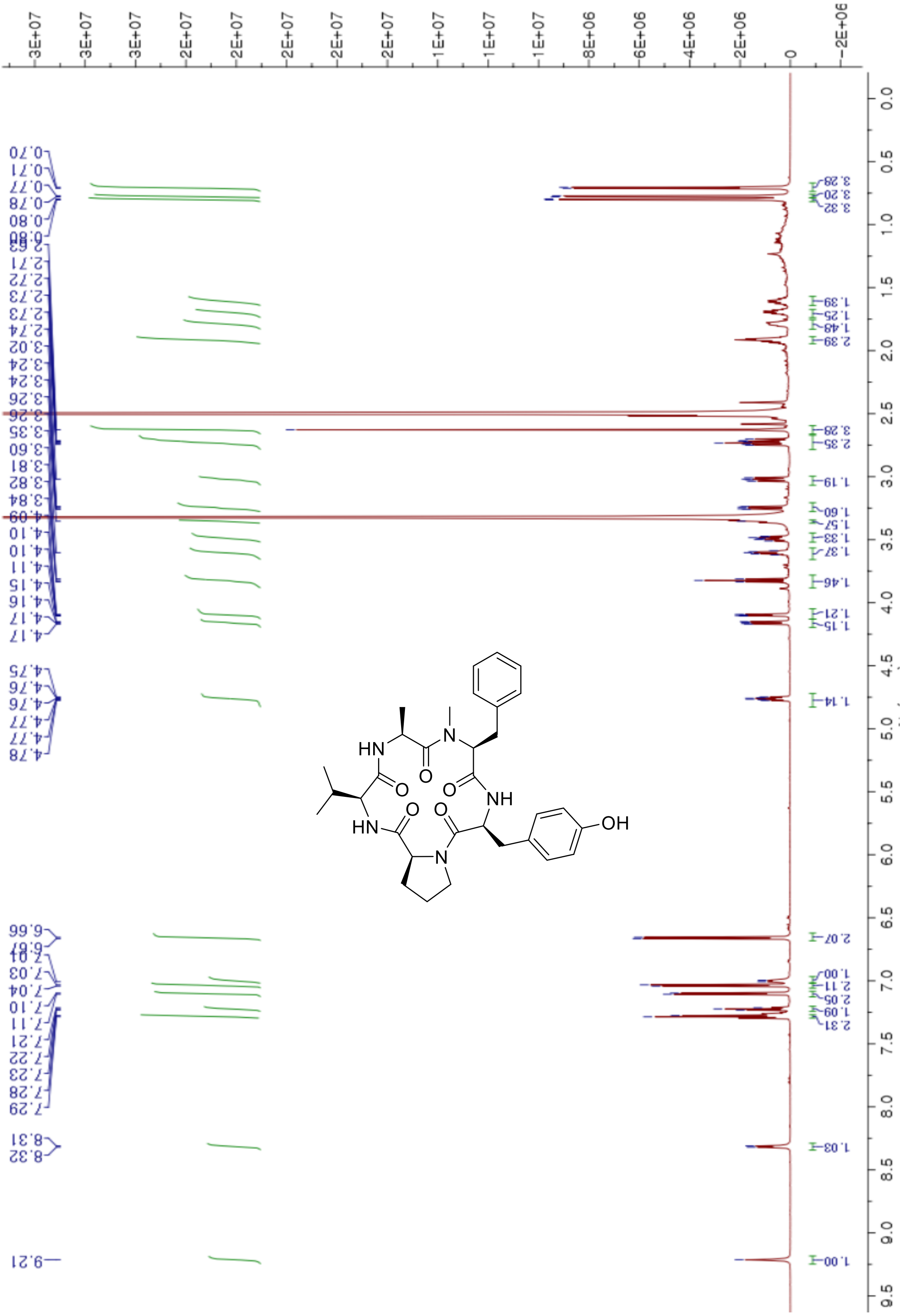

Figure S8. The ^{1}H NMR spectrum of JG002CPB (**2**) (800MHz, DMSO-d_6)

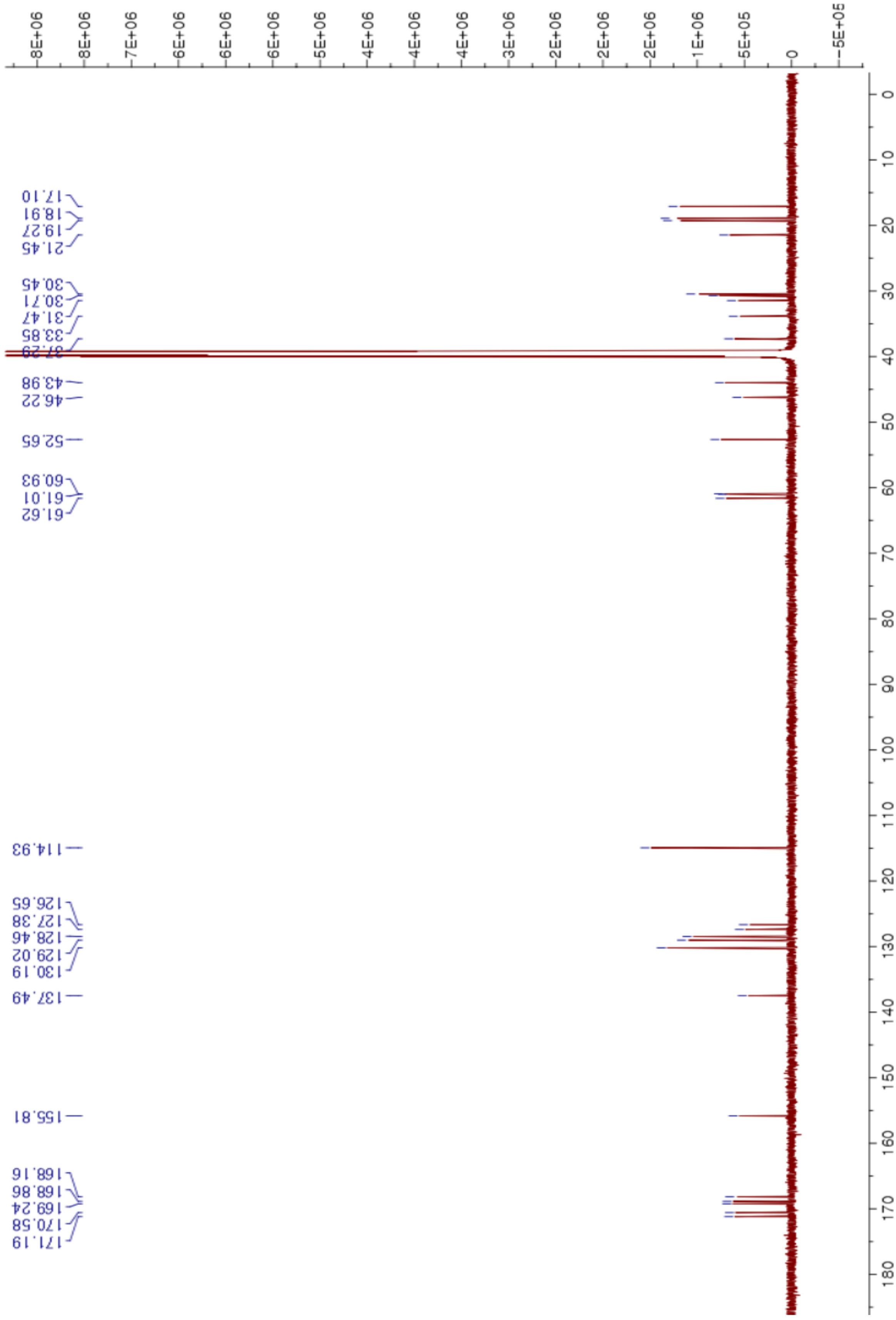

Figure S9. The ^{13}C NMR spectrum of JG002CPB (**2**) (200MHz, DMSO-d_6)

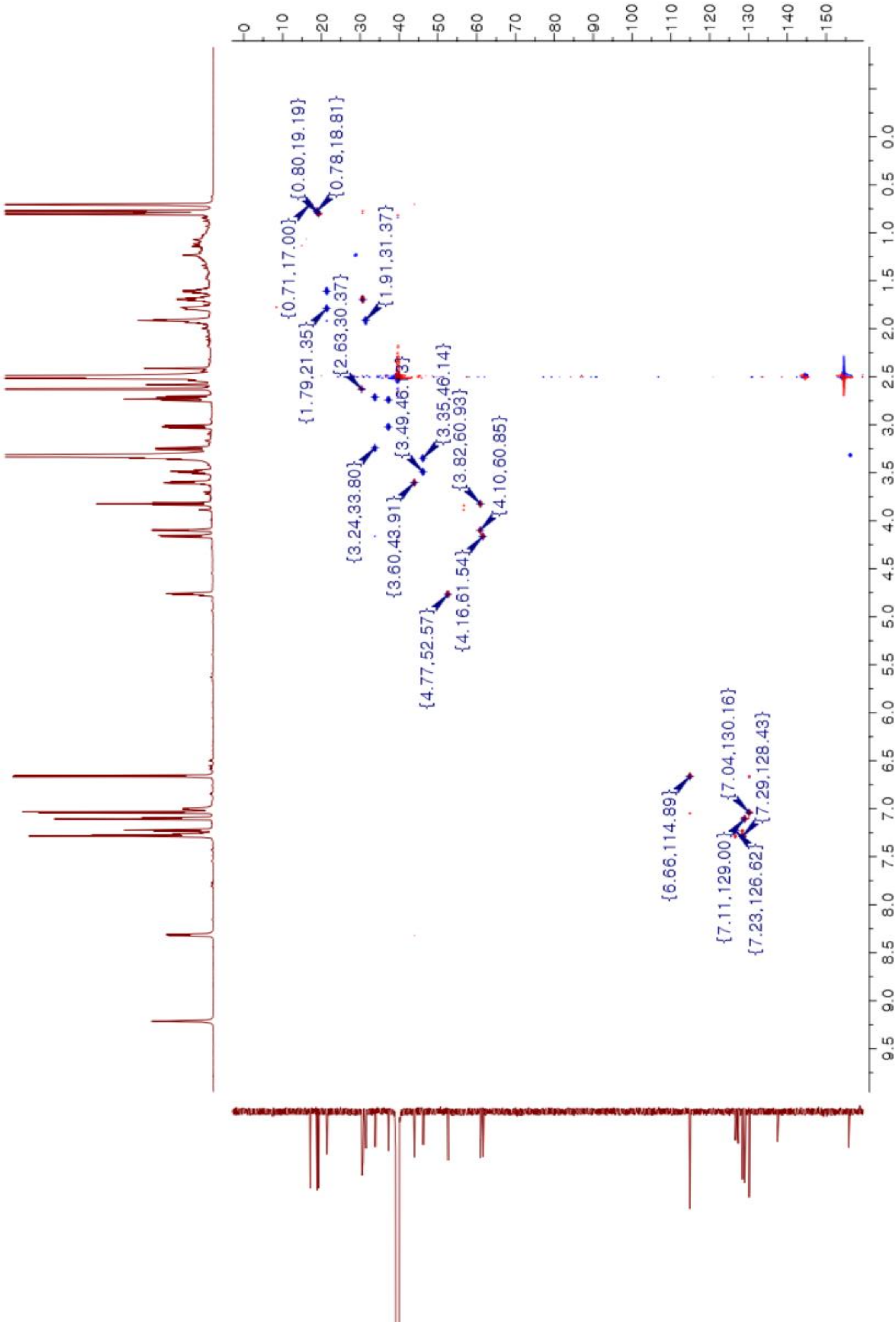

Figure S10. The HSQC spectrum of JG002CPB (**2**) (800MHz, DMSO-d_6)

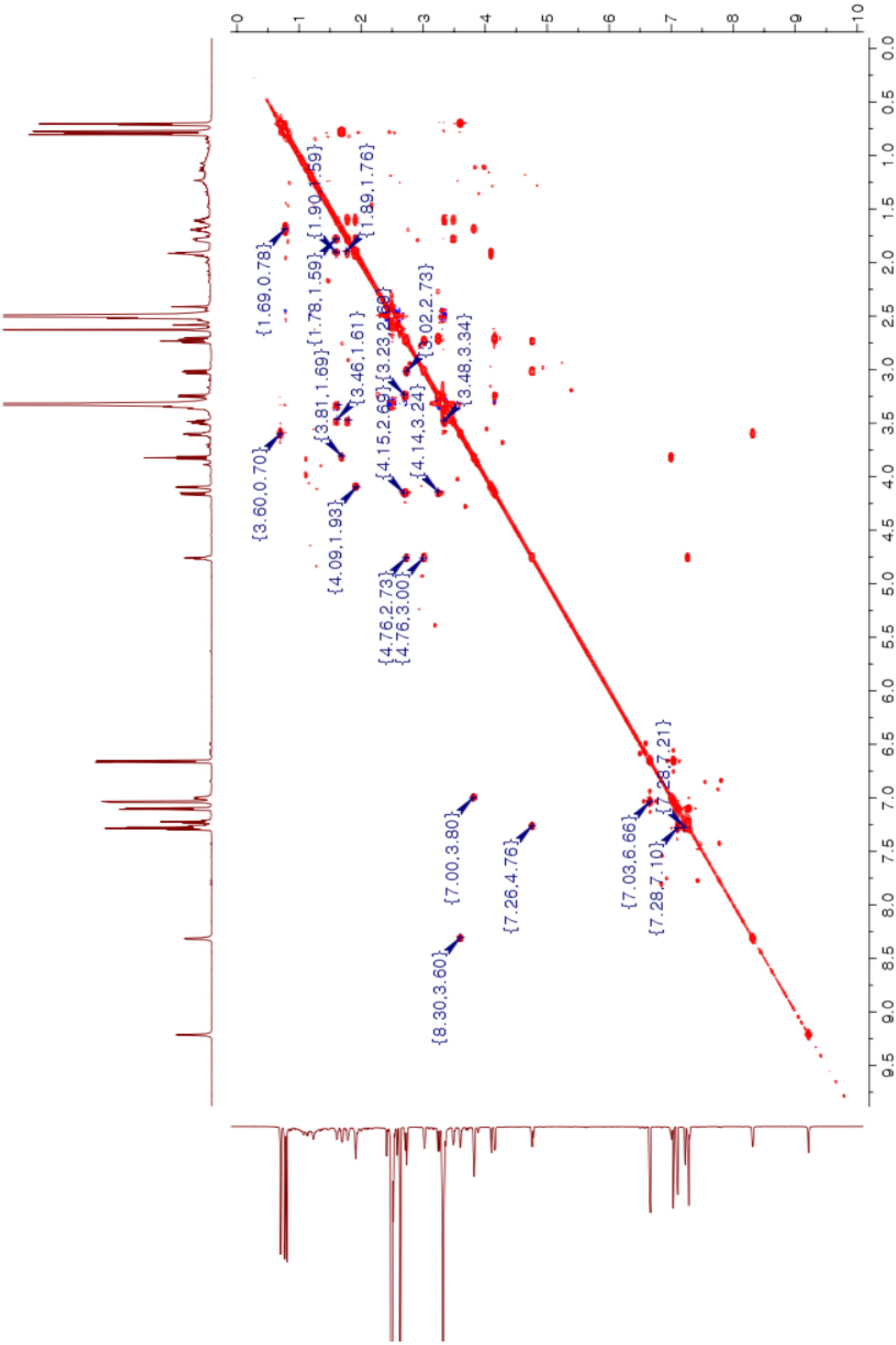

Figure S11. The COSY spectrum of JG002CPB (**2**) (800MHz, DMSO-d_6)

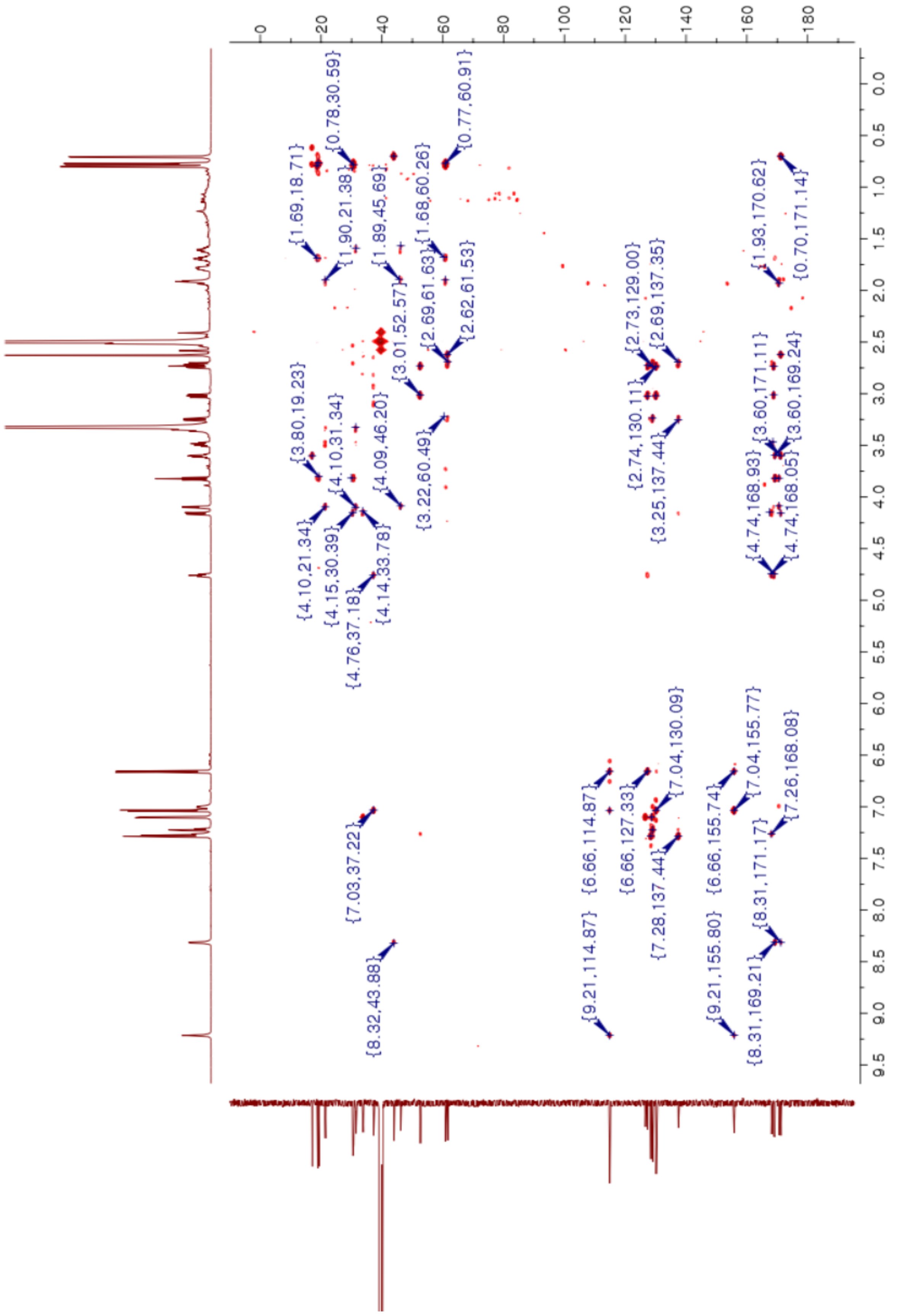

Figure S12. The HMBC spectrum of JG002CPB (**2**) (800MHz, DMSO-d_6)

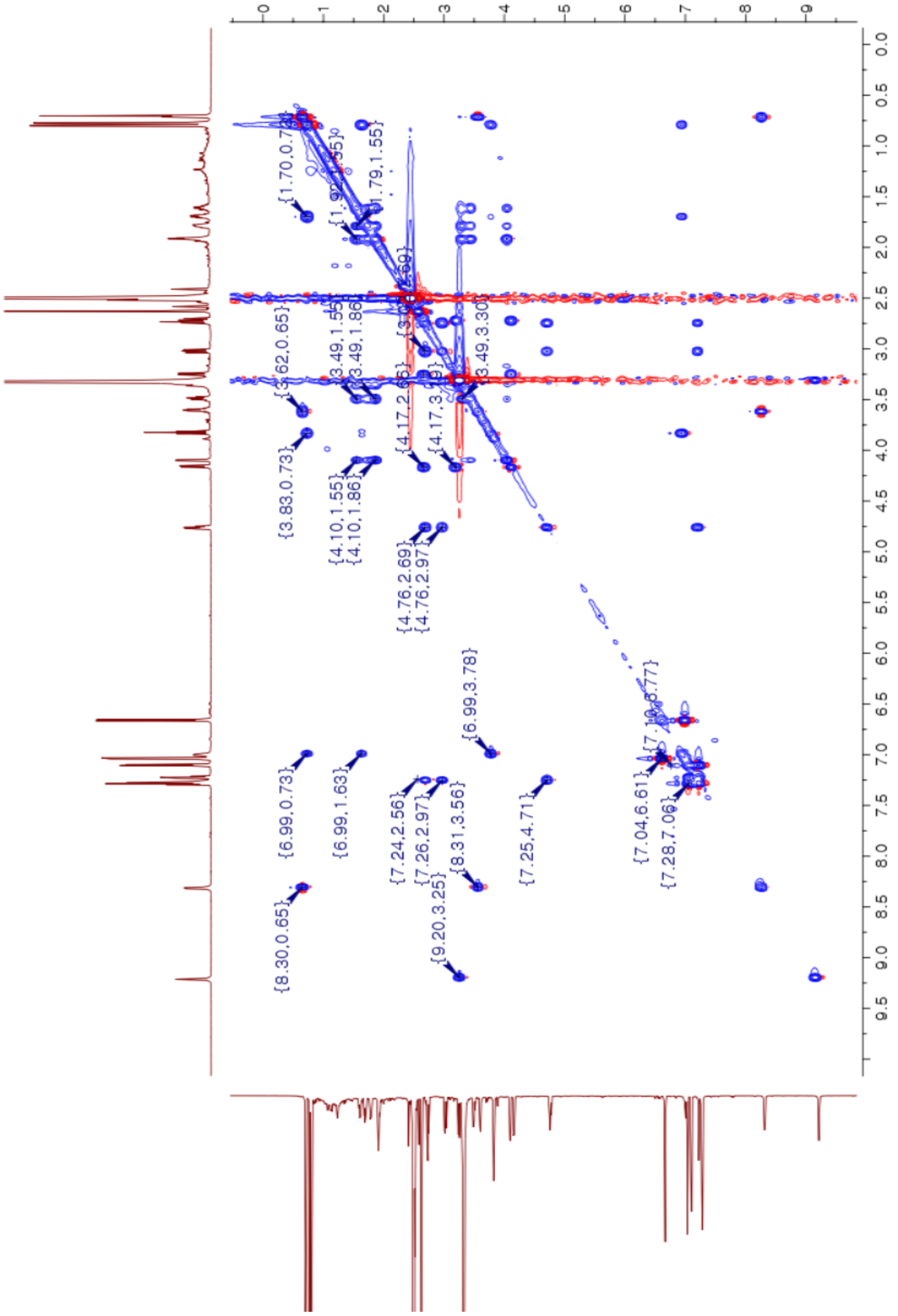

Figure S13. The TOCSY spectrum of JG002CPB (**2**) (600MHz, DMSO-*d*6)

[Elemental Composition]
Data : FAB-S653 Date : 07-Jun-2019 14:33
Sample: 2 [JG002-RF2RP10]
Note : m-NBA
Inlet : Direct Ion Mode : FAB+
RT : 0.50 min Scan#: (18,24)
Elements : C 100/0, H 100/0, N 10/0, O 10/0
Mass Tolerance : 20ppm, 5mmu if m/z < 250, 10mmu if m/z > 500
Unsaturation (U.S.) : -0.5 - 50.0

Observed m/z	Int%	Err [ppm / mmu]	U.S.	Composition
592.3133	83.8	+0.5 / +0.3	27.0	C 46 H 40
		-9.4 / -5.6	23.5	C 38 H 38 N 7
		+11.8 / +7.0	24.0	C 37 H 36 N 8
		-11.7 / -6.9	23.0	C 40 H 40 N 4 O
		+9.6 / +5.7	23.5	C 39 H 38 N 5 O
		-13.9 / -8.3	22.5	C 42 H 42 N O 2
		+7.3 / +4.3	23.0	C 41 H 40 N 2 O 2
		-2.6 / -1.6	19.5	C 33 H 38 N 9 O 2
		-4.9 / -2.9	19.0	C 35 H 40 N 6 O 3
		+16.3 / +9.7	19.5	C 34 H 38 N 7 O 3
		-7.2 / -4.2	18.5	C 37 H 42 N 3 O 4
		+14.1 / +8.3	19.0	C 36 H 40 N 4 O 4
		-9.4 / -5.6	18.0	C 39 H 44 O 5
		+11.8 / +7.0	18.5	C 38 H 42 N O 5
		+1.9 / +1.1	15.0	C 30 H 40 N 8 O 5
		-0.4 / -0.2	14.5	C 32 H 42 N 5 O 6
		-2.6 / -1.6	14.0	C 34 H 44 N 2 O 7
		-12.5 / -7.4	10.5	C 26 H 42 N 9 O 7
		+8.7 / +5.1	11.0	C 25 H 40 N 10 O 7
		+16.3 / +9.7	14.0	C 35 H 44 O 8
		-14.8 / -8.8	10.0	C 28 H 44 N 6 O 8
		+6.4 / +3.8	10.5	C 27 H 42 N 7 O 8
		+4.2 / +2.5	10.0	C 29 H 44 N 4 O 9
		+1.9 / +1.1	9.5	C 31 H 46 N O 10
		-8.0 / -4.7	6.0	C 23 H 44 N 8 O 10
		+13.2 / +7.8	6.5	C 22 H 42 N 9 O 10

[Theoretical Ion Distribution] Page: 1
Molecular Formula : C32 H42 N5 O6
(m/z 592.3135, MW 592.7154, U.S. 14.5)
Base Peak : 592.3135, Averaged MW : 592.7113(a), 592.7120(w)

m/z	INT.	
592.3135	100.0000	**
593.3166	37.6568	**********************
594.3193	8.0917	*****
595.3220	1.2681	*
596.3246	0.1590	
597.3271	0.0167	
598.3296	0.0015	
599.3322	0.0001	

Figure S14. The HRFABMS data of JG002CPB (**2**)

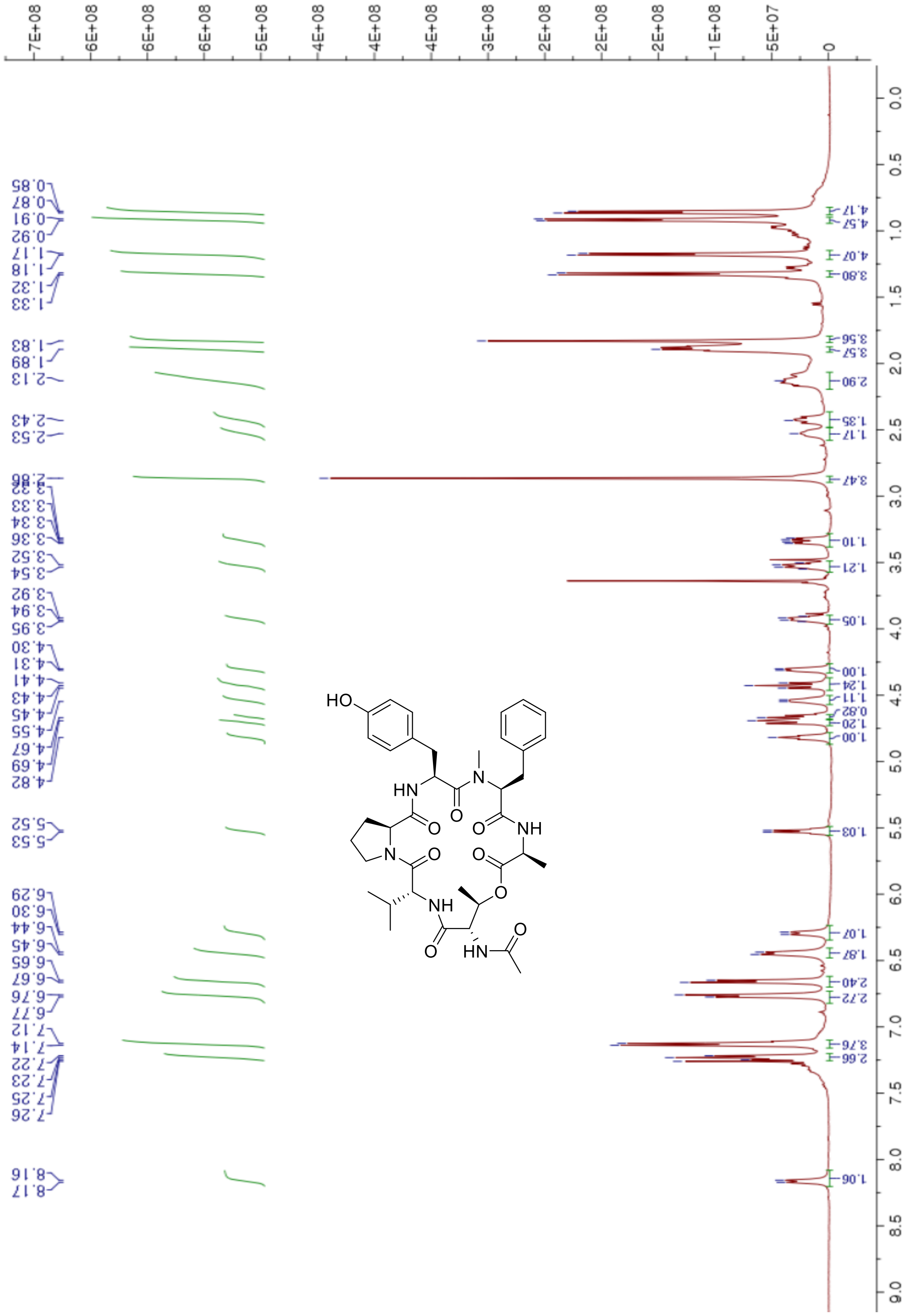

Figure S15. The ¹H NMR spectrum of FJ120DPA (**3**) (500MHz, $CDCl_3$)

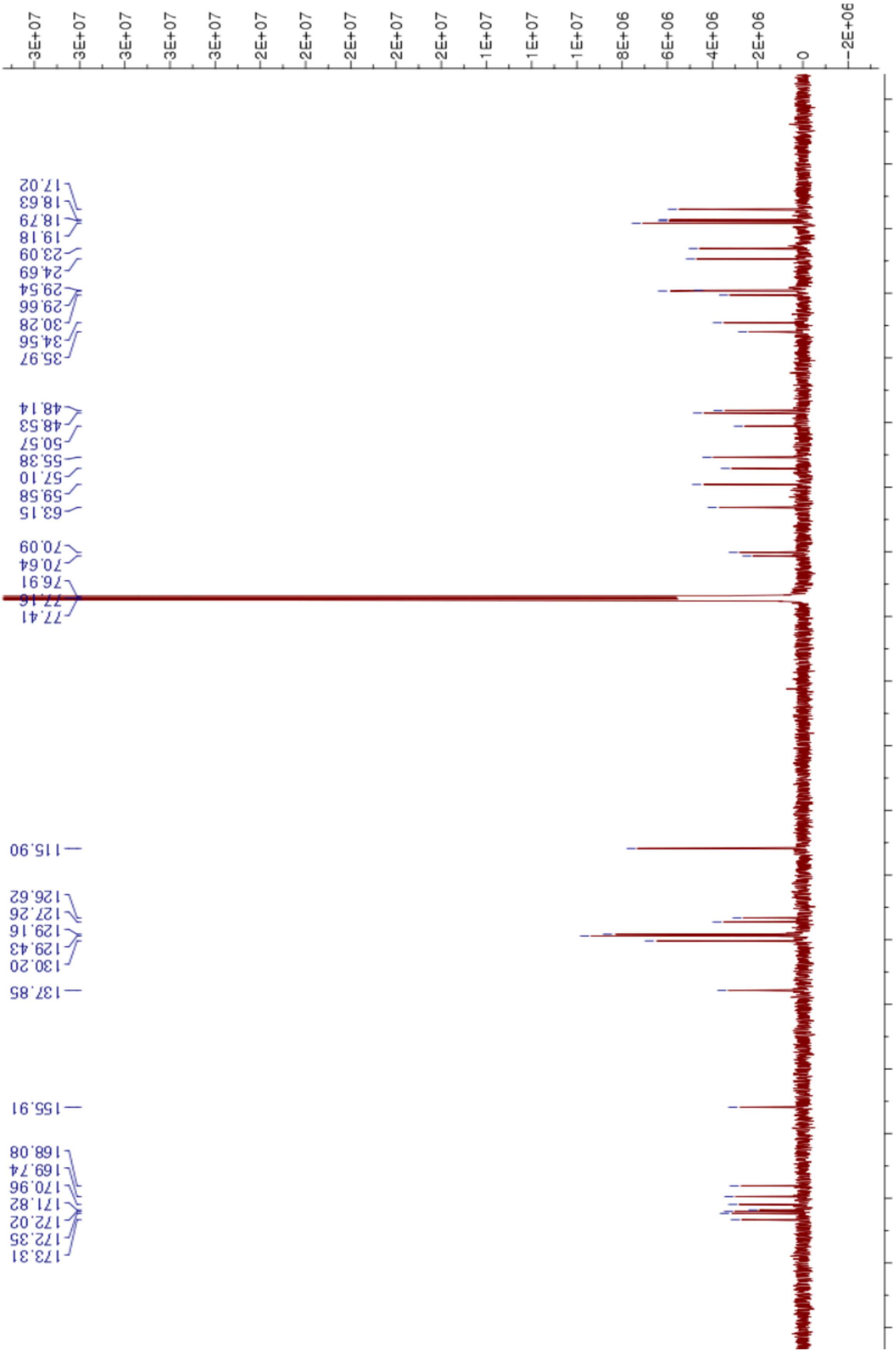

Figure S16. The ^{13}C NMR spectrum of FJ120DPA (**3**) (125MHz, $CDCl_3$)

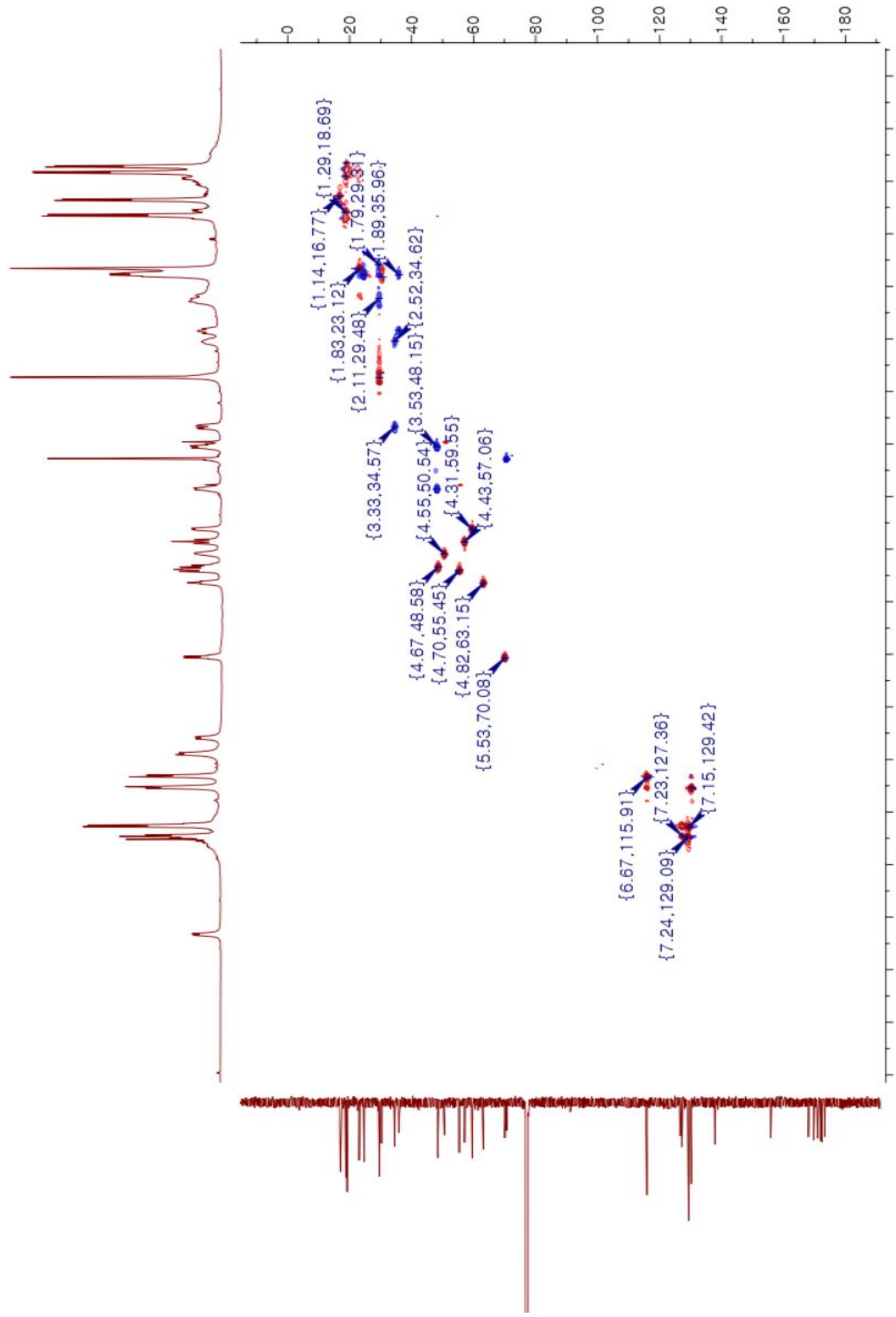

Figure S17. The HSQC spectrum of FJ120DPA (**3**) (500MHz, $CDCl_3$)

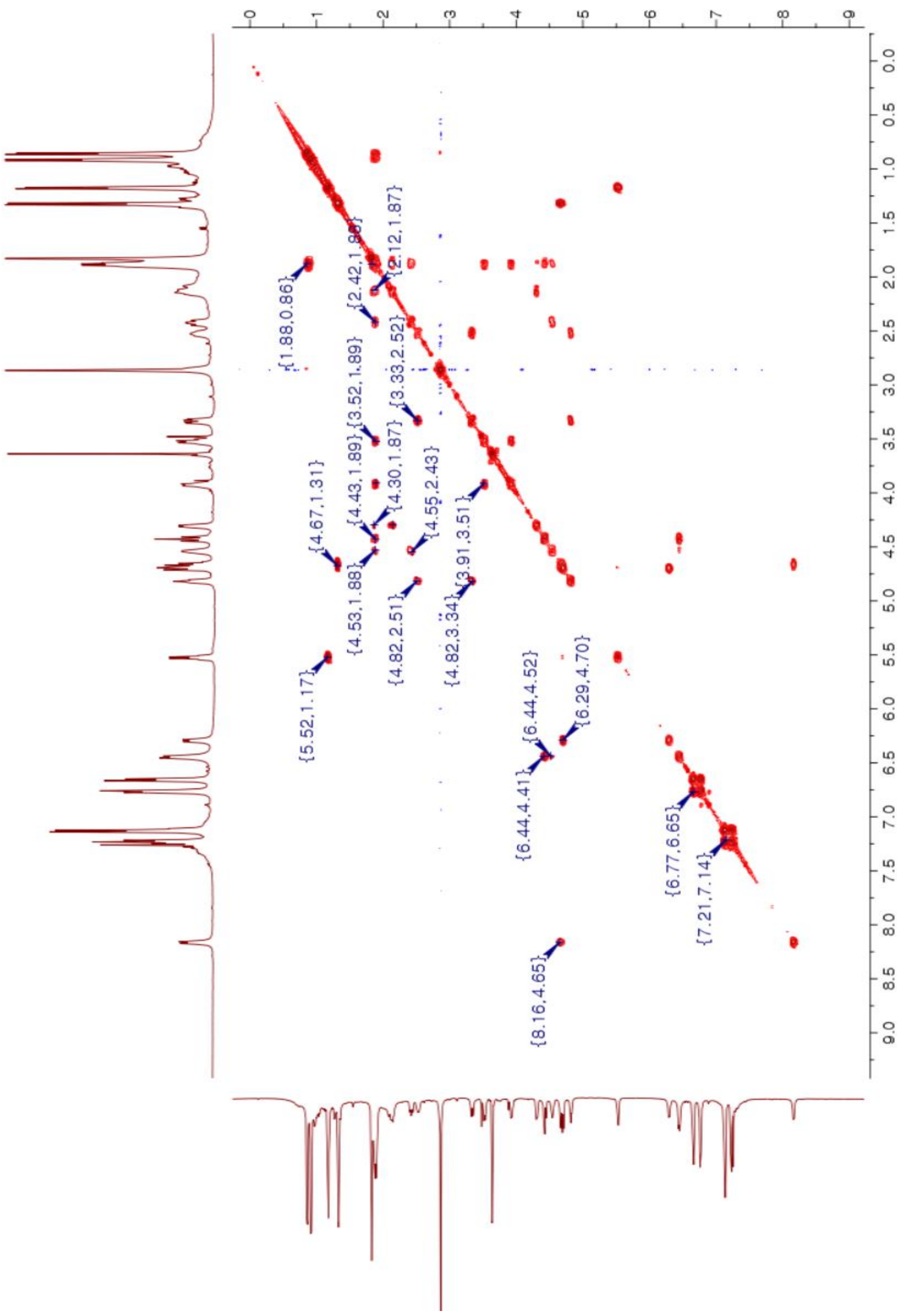

Figure S18. The COSY spectrum of FJ120DPA (**3**) (500MHz, $CDCl_3$)

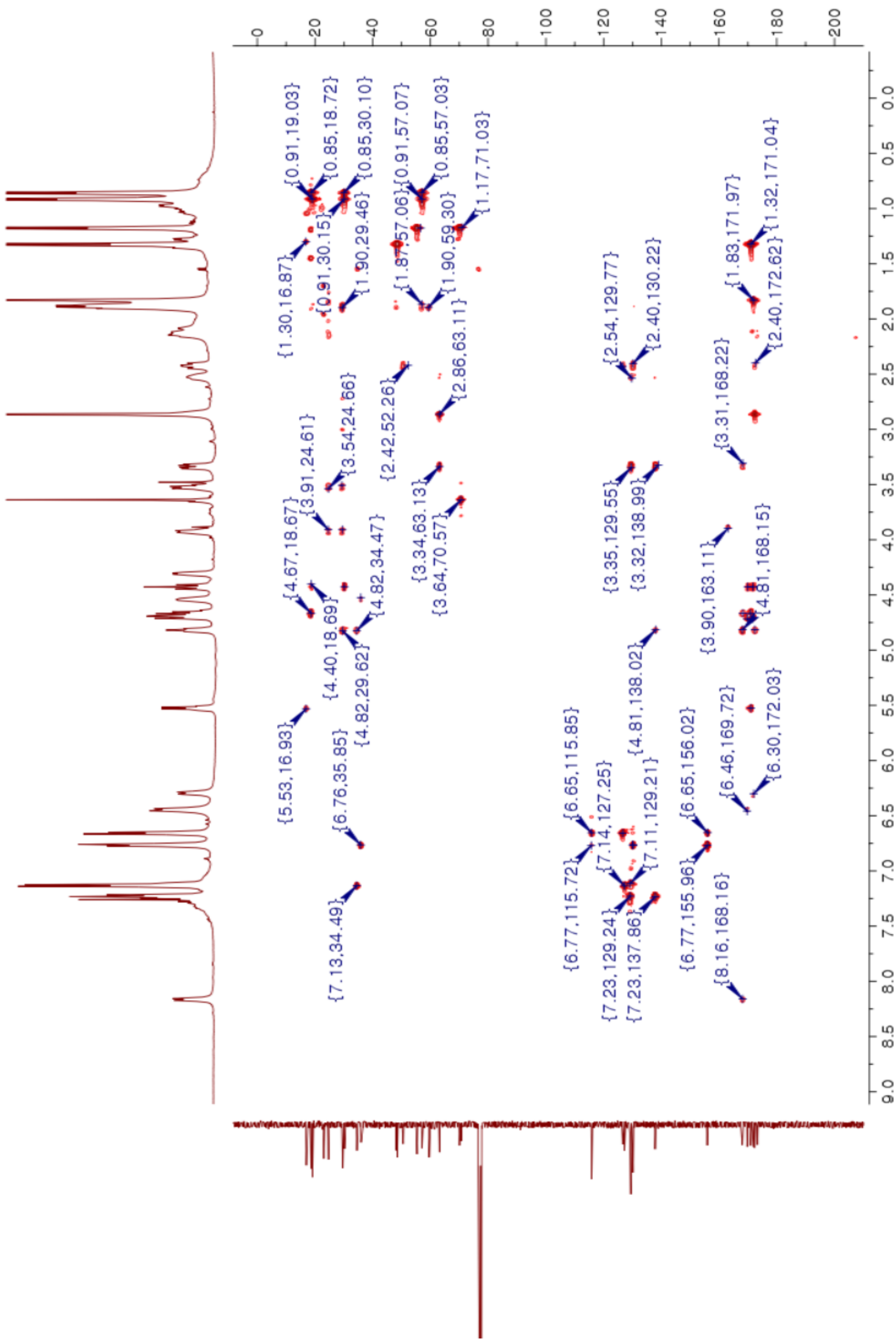

Figure S19. The HMBC spectrum of FJ120DPA (**3**) (500MHz, $CDCl_3$)

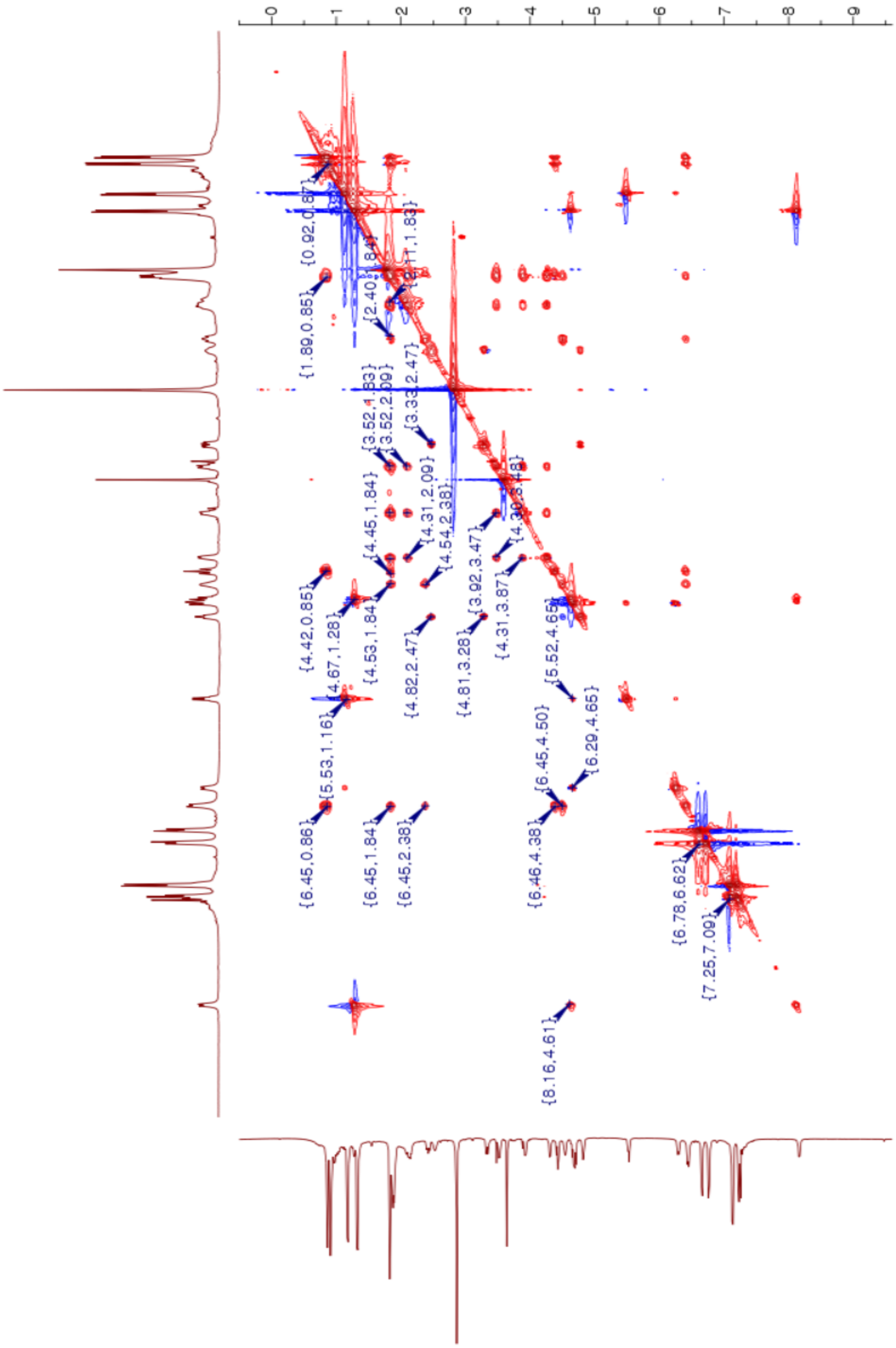

Figure S20. The TOCSY spectrum of FJ120DPA (**3**) (500MHz, $CDCl_3$)

[Elemental Composition] Page: 1

Data : FJ120-A-C38H50N6O9 Date : 29-May-2012 10:03
Sample: -
Note : -
Inlet : Direct Ion Mode : FAB+
RT : 0.23 min Scan#: (5,6)
Elements : C 38/0, H 51/1, O 9/1, N 6/1
Mass Tolerance : 1000ppm, 1mmu if m/z < 1, 3mmu if m/z > 3
Unsaturation (U.S.) : -0.5 - 200.0

Observed m/z	Int%	Err[ppm / mmu]	U.S.	Composition
734.3661	10.2	+2.9 / +2.2	17.0	C 38 H 50 O 9 N 6
735.3721	70.0	+0.5 / +0.4	16.5	C 38 H 51 O 9 N 6
736.3742	32.2			
757.3534	100.0			
758.3586	44.1			
759.3664	12.5			

[Theoretical Ion Distribution] Page: 1

Molecular Formula : C38 H51 O9 N6
(m/z 735.3718, MW 735.8577, U.S. 16.5)
Base Peak : 735.3718, Averaged MW : 735.8603 (a), 735.8610 (w)

m/z	INT.	
735.3718	100.0000	**
736.3749	45.5769	***************************
737.3777	11.9514	*******
738.3803	2.2923	*
739.3830	0.3533	
740.3855	0.0460	
741.3881	0.0052	
742.3906	0.0005	

Figure S21. The HRFABMS data of FJ120DPA (**3**)

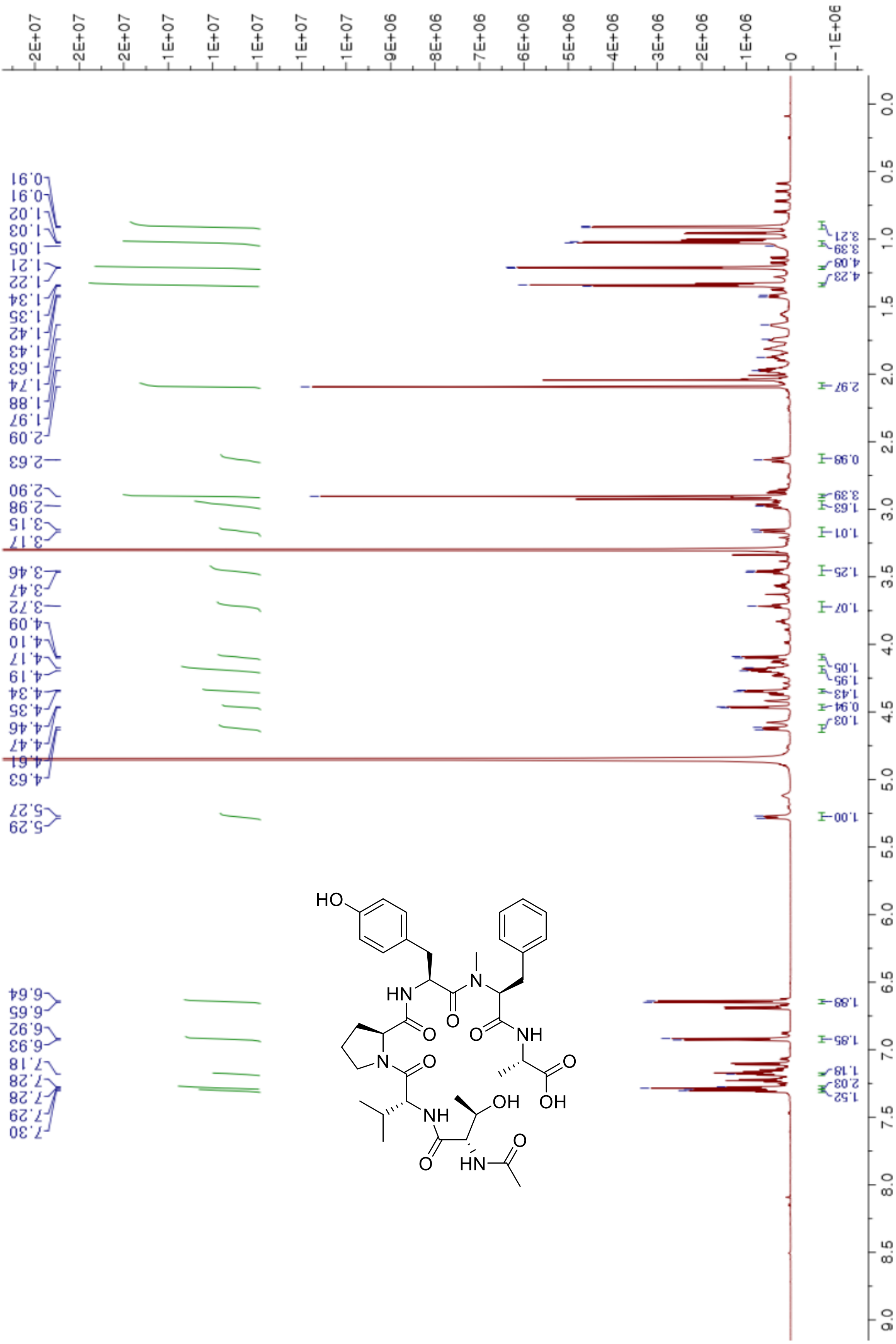

Figure S22. The ^{1}H NMR spectrum of FJ120DPB (**4**) (800MHz, CD_3OD)

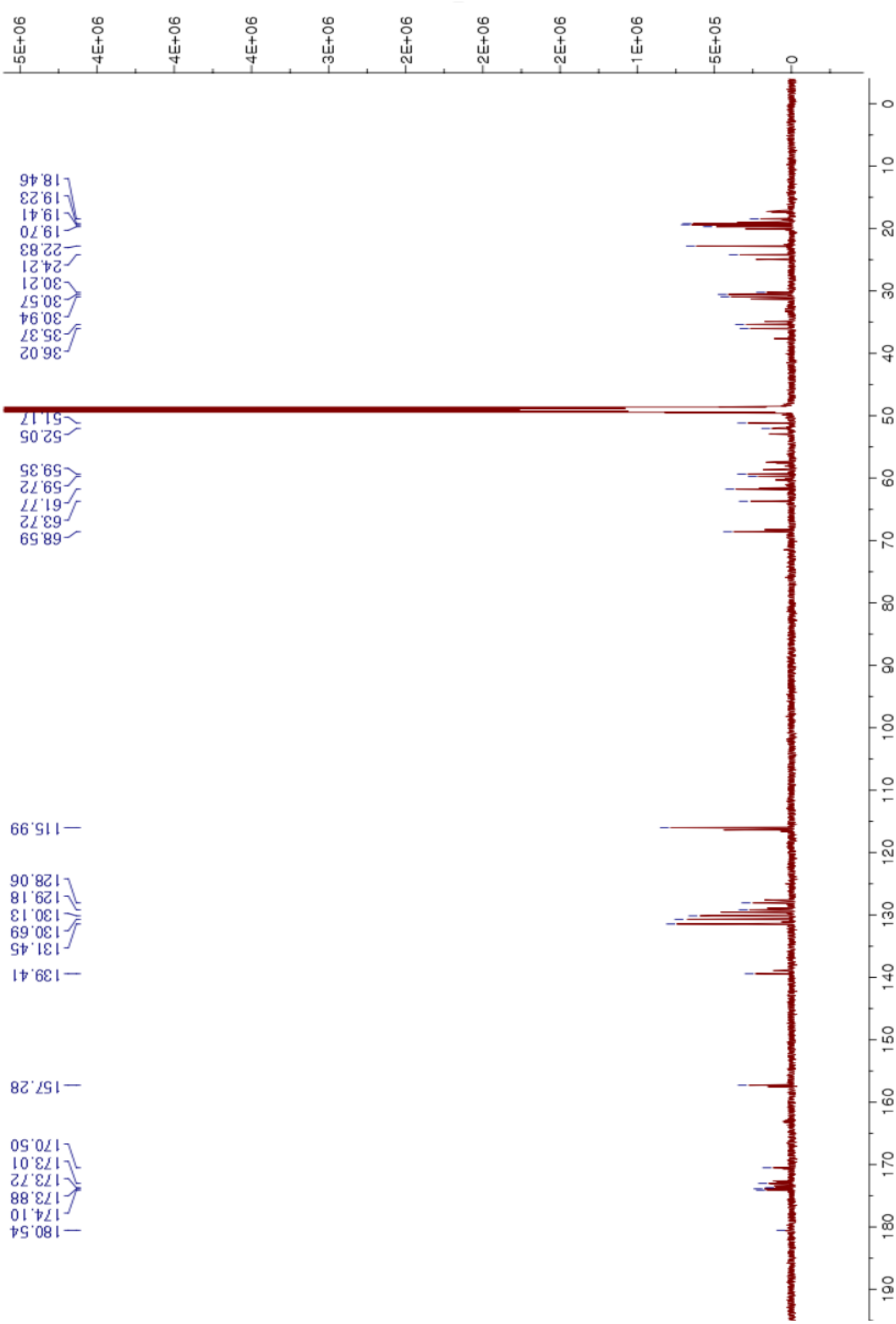

Figure S23. The ^{13}C NMR spectrum of FJ120DPB (**4**) (200MHz, CD_3OD)

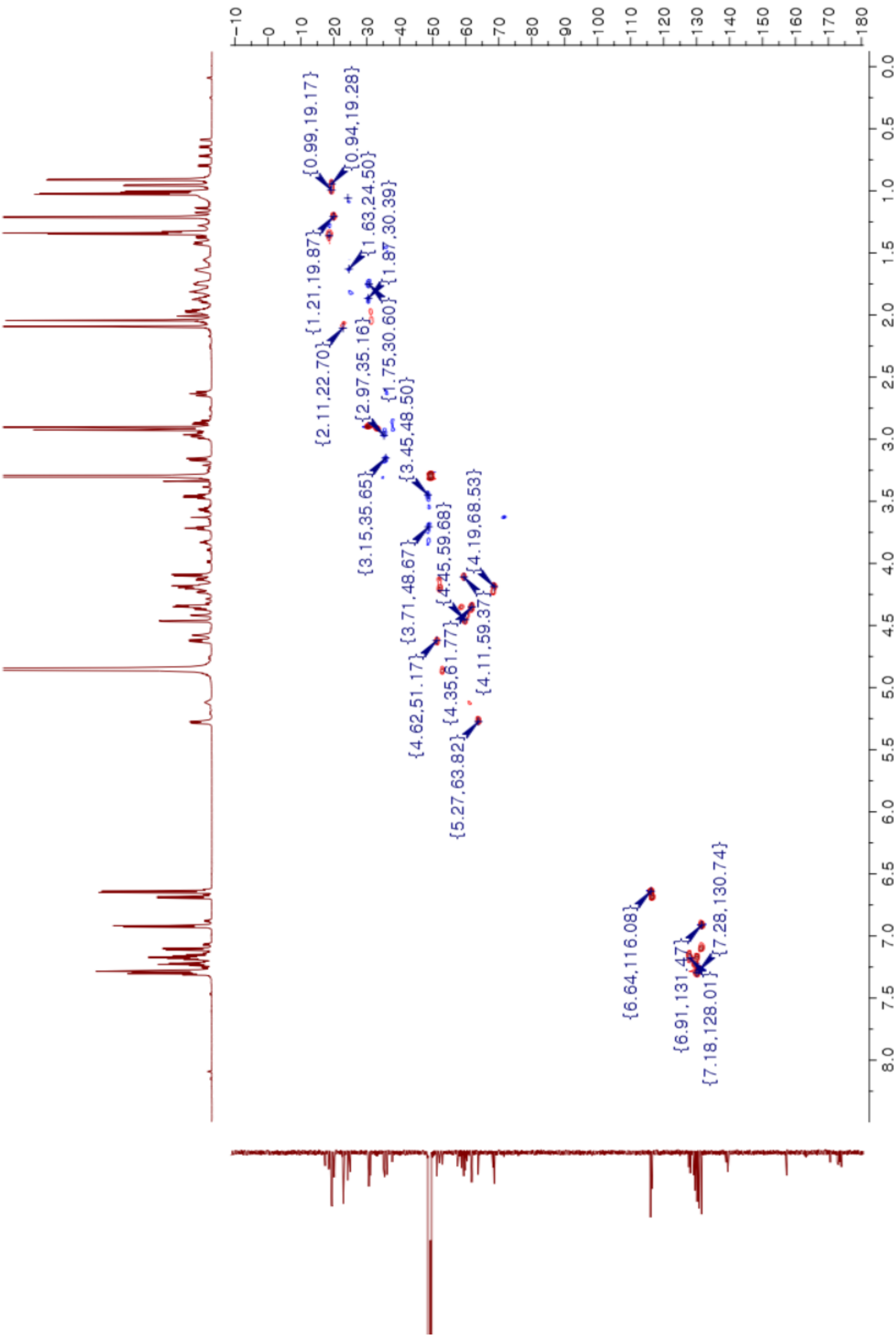

Figure S24. The HSQC spectrum of FJ120DPB (**4**) (500MHz, CD_3OD)

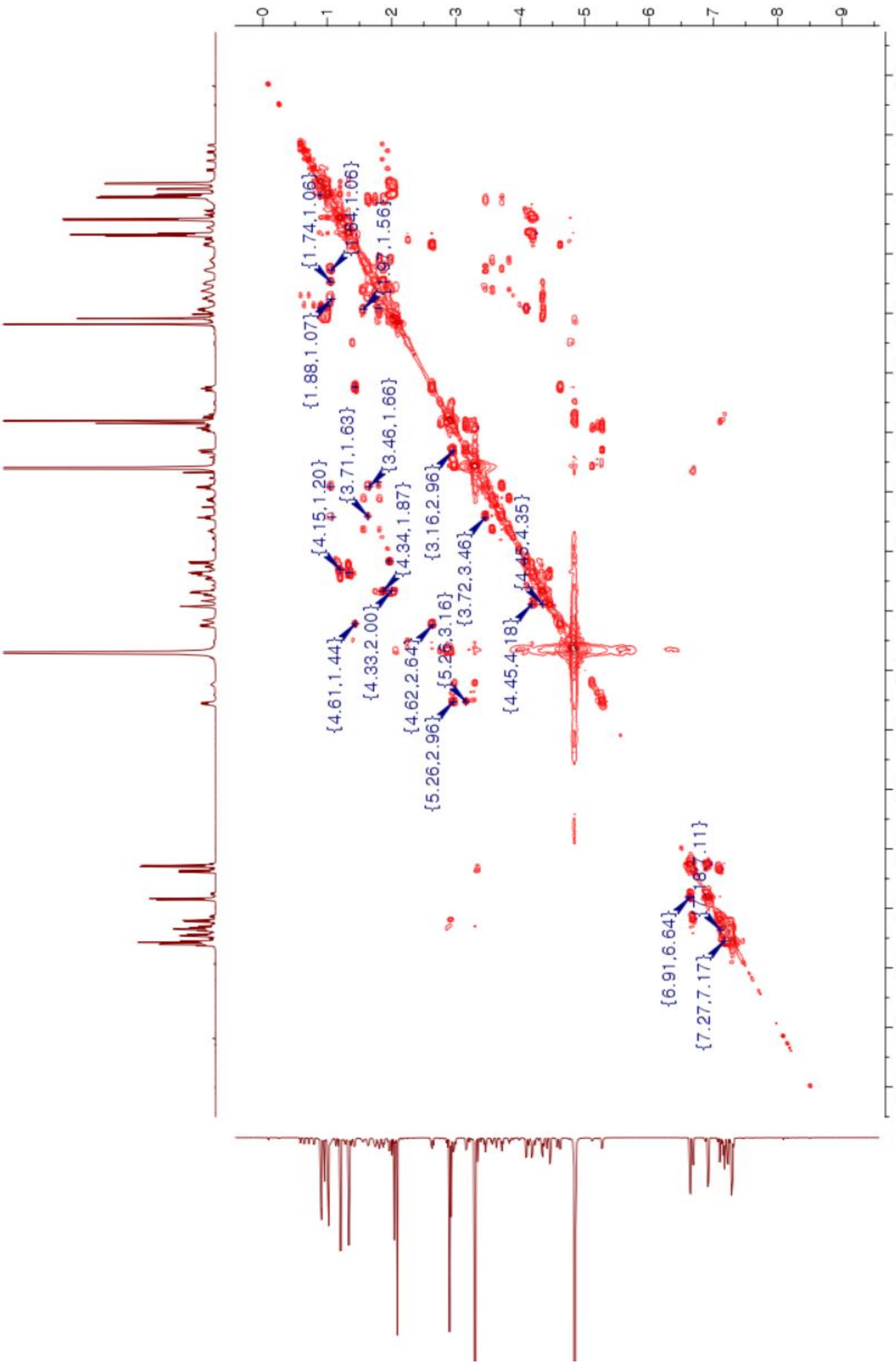

Figure S25. The COSY spectrum of FJ120DPB (**4**) (600MHz, CD_3OD)

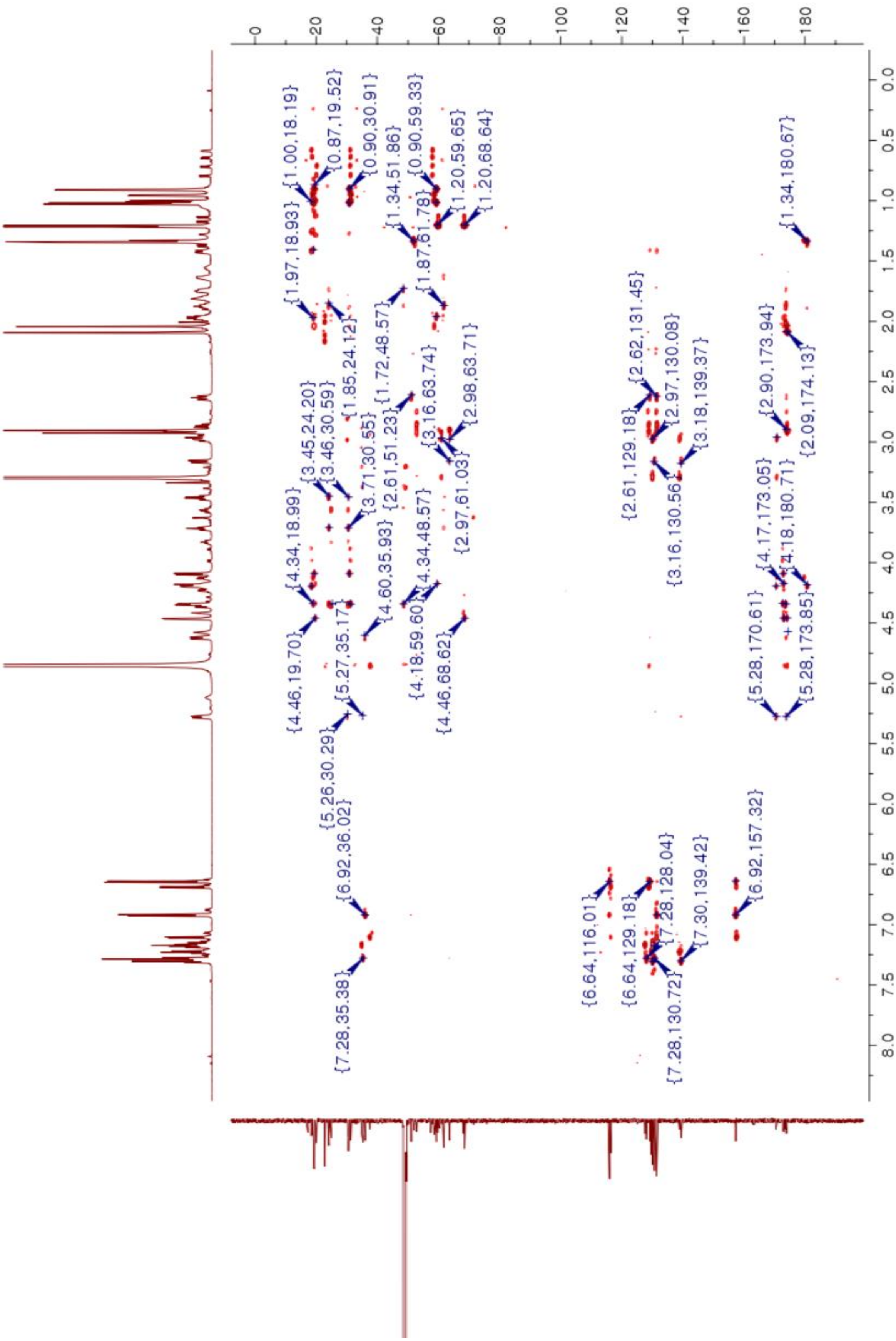

Figure S26. The HMBC spectrum of FJ120DPB (**4**) (800MHz, CD_3OD)

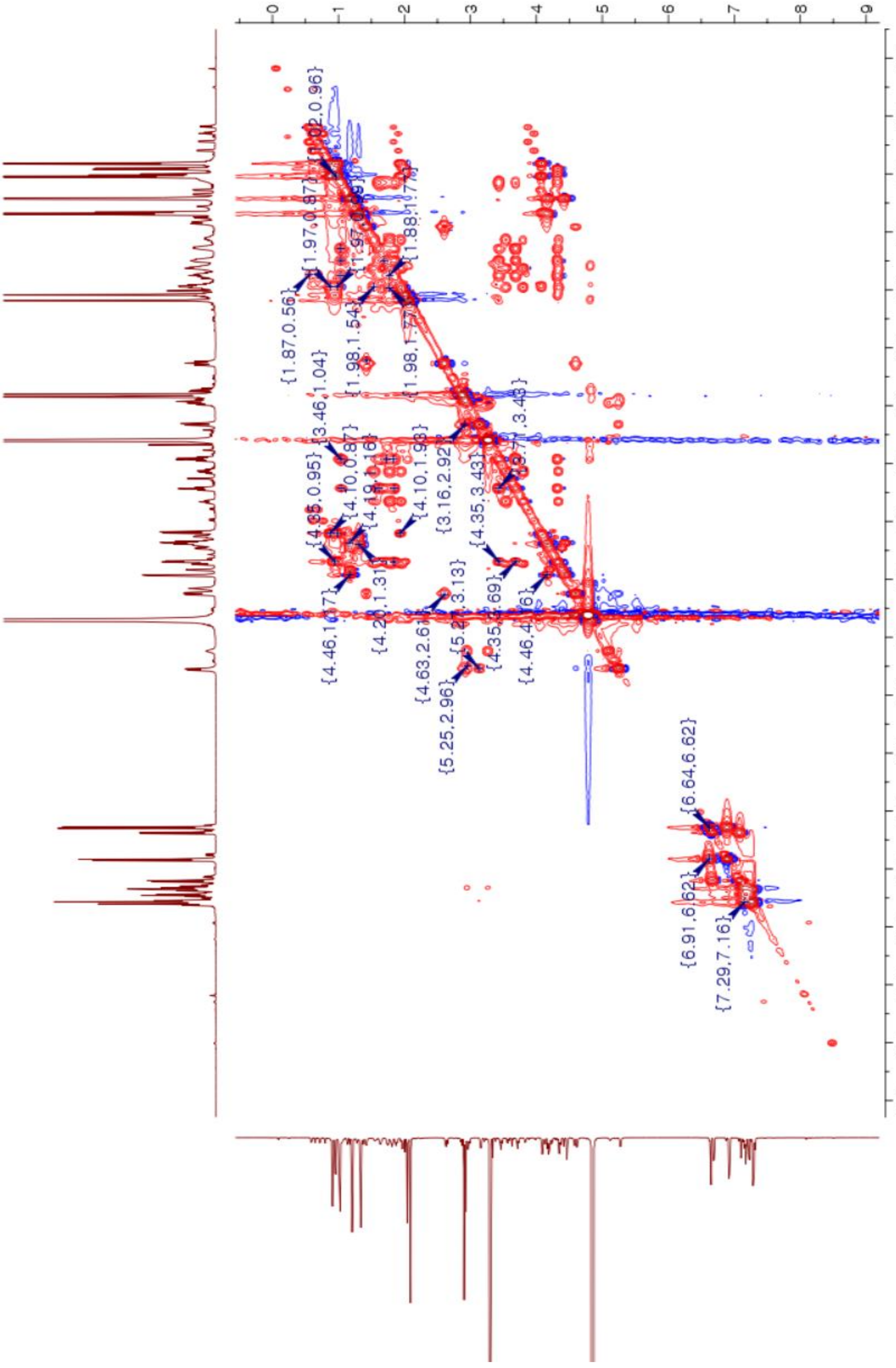

Figure S27. The TOCSY spectrum of FJ120DPB (**4**) (600MHz, CD_3OD)

[Elemental Composition]   Page: 1

Data : FJ120-rf1-rp7a-rp10-HRFAB   Date : 29-Jun-2012 14:43
Sample: -
Note : -
Inlet : Direct   Ion Mode : FAB+
RT : 0.23 min   Scan#: (5,6)
[illegible]lements : C 38/0, H 52/1, O 10/1, N 6/1, Na 1/1
[illegible]ass Tolerance : 1000ppm, 1mmu if m/z < 1, 3mmu if m/z > 3
Unsaturation (U.S.) : -0.5 - 200.0

Observed m/z	Int%	Err[ppm / mmu]	U.S.	Composition
658.3195	19.4	+0.8 / +0.5	10.0	C 31 H 47 O 10 N 4 Na
		-3.3 / -2.1	14.5	C 34 H 45 O 7 N 5 Na
664.3356	13.3	+3.1 / +2.1	12.0	C 35 H 49 O 9 N 2 Na
		-0.9 / -0.6	16.5	C 38 H 47 O 6 N 3 Na
		+1.1 / +0.7	17.0	C 36 H 45 O 5 N 6 Na
701.3016	13.9	-3.0 / -2.1	16.0	C 35 H 44 O 9 N 5 Na
703.3270	10.7			
729.3600	15.0	+1.6 / +1.2	15.5	C 37 H 50 O 8 N 6 Na
747.3112	31.7			
748.3127	15.7			
769.2896	30.4			
770.2930	14.3			
773.3265	14.7			
774.3479	11.9			
775.3646	88.9	+0.5 / +0.4	15.5	C 38 H 52 O 10 N 6 Na
776.3694	38.9			
777.3693	12.5			
785.2599	32.3			
786.2603	14.4			
789.3073	10.2			
790.3256	12.9			
791.3314	100.0			
792.3474	49.5			
793.3506	16.9			
797.3503	22.0			
798.3508	10.1			

[Theoretical Ion Distribution]   Page: 1

Molecular Formula : C38 H52 O10 N6 Na
(m/z 775.3643, MW 775.8548, U.S. 15.5)
Base Peak : 775.3643, Averaged MW : 775.8573(a), 775.8580(w)

```
  m/z        INT.
775.3643 100.0000 **********************************************************
776.3674  45.6300 **************************
777.3701  12.1760 *******
778.3728   2.3900 *
779.3754   0.3784
780.3780   0.0508
781.3805   0.0059
782.3830   0.0006
```

Figure S28. The HRFABMS data of FJ120DPB (**4**)

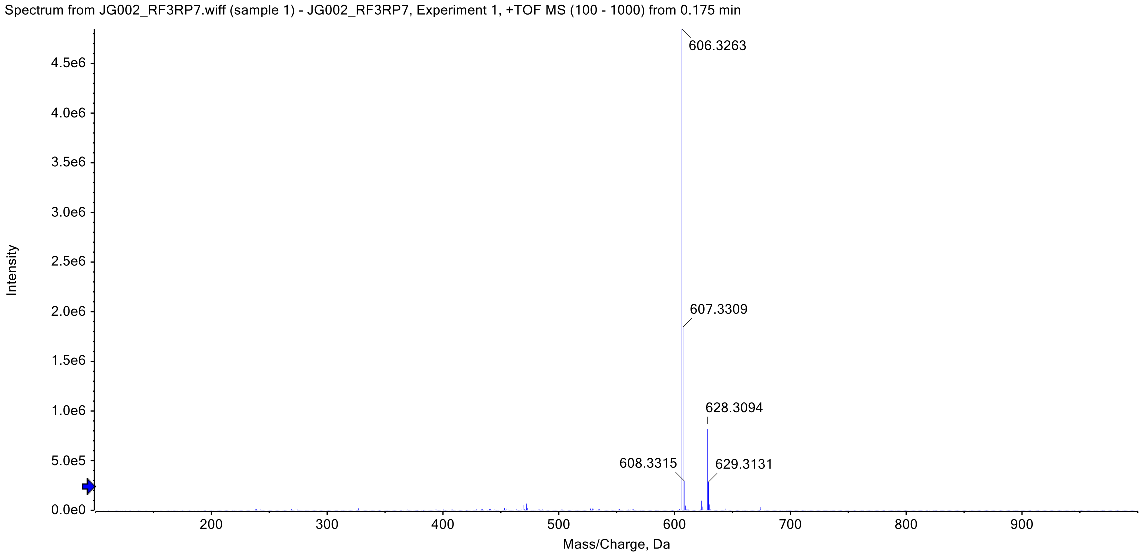

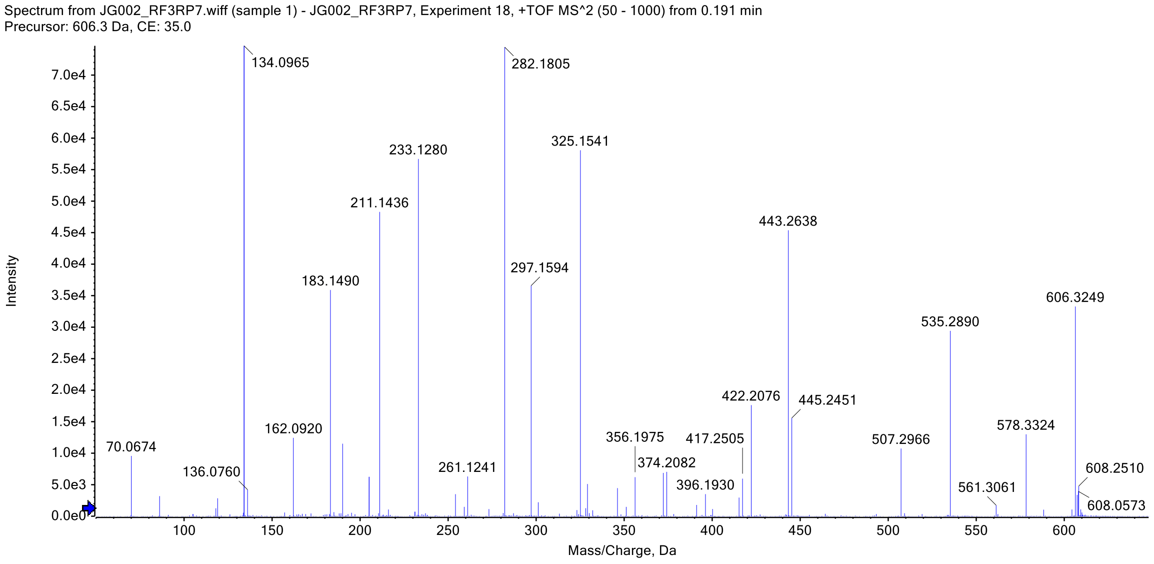

Figure S29. The high-resolution LC/MS-MS fragmentation analysis of JG002CPA (**1**)

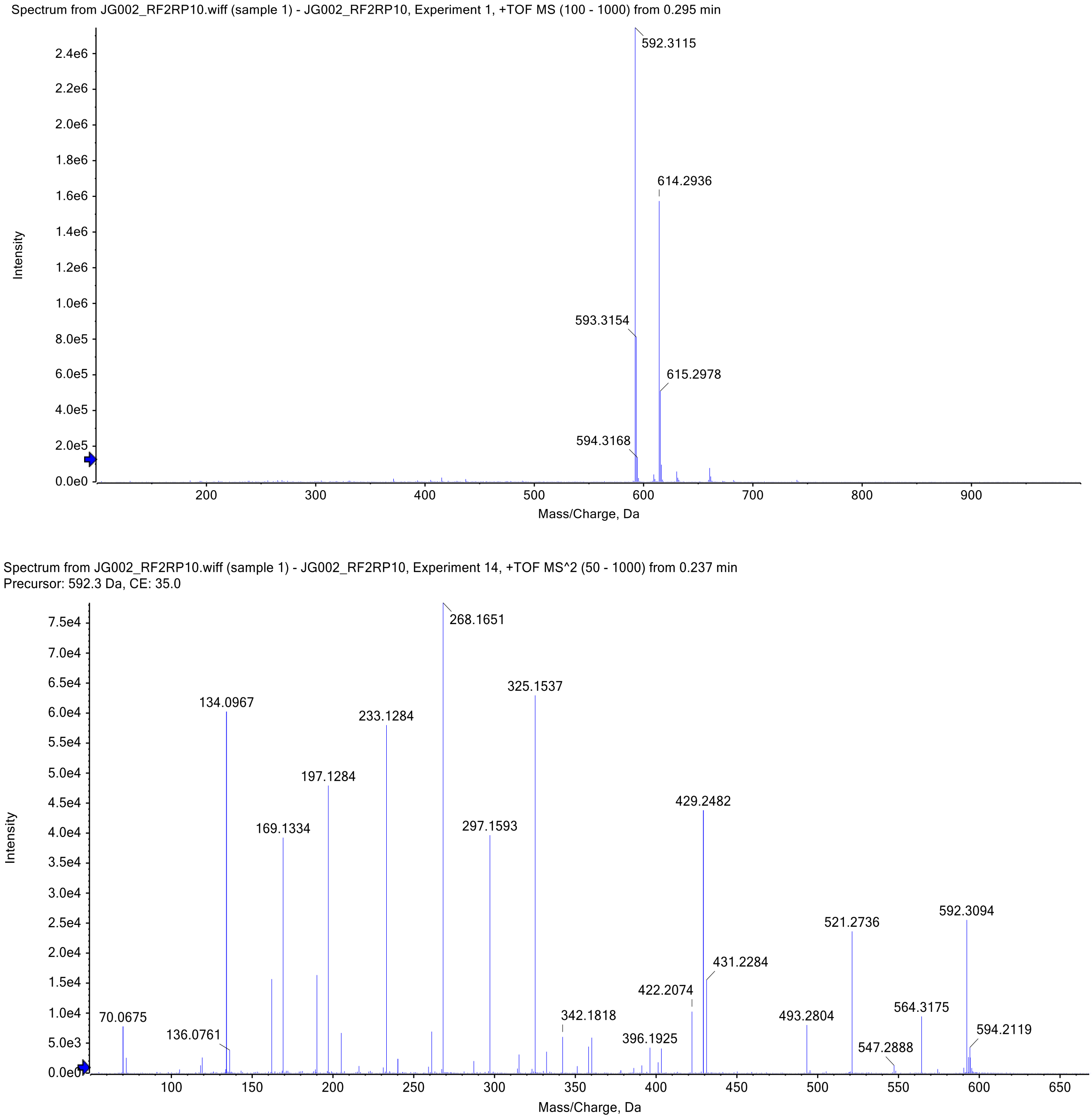

Figure S30. The high-resolution LC/MS-MS fragmentation analysis of JG002CPB (**2**)

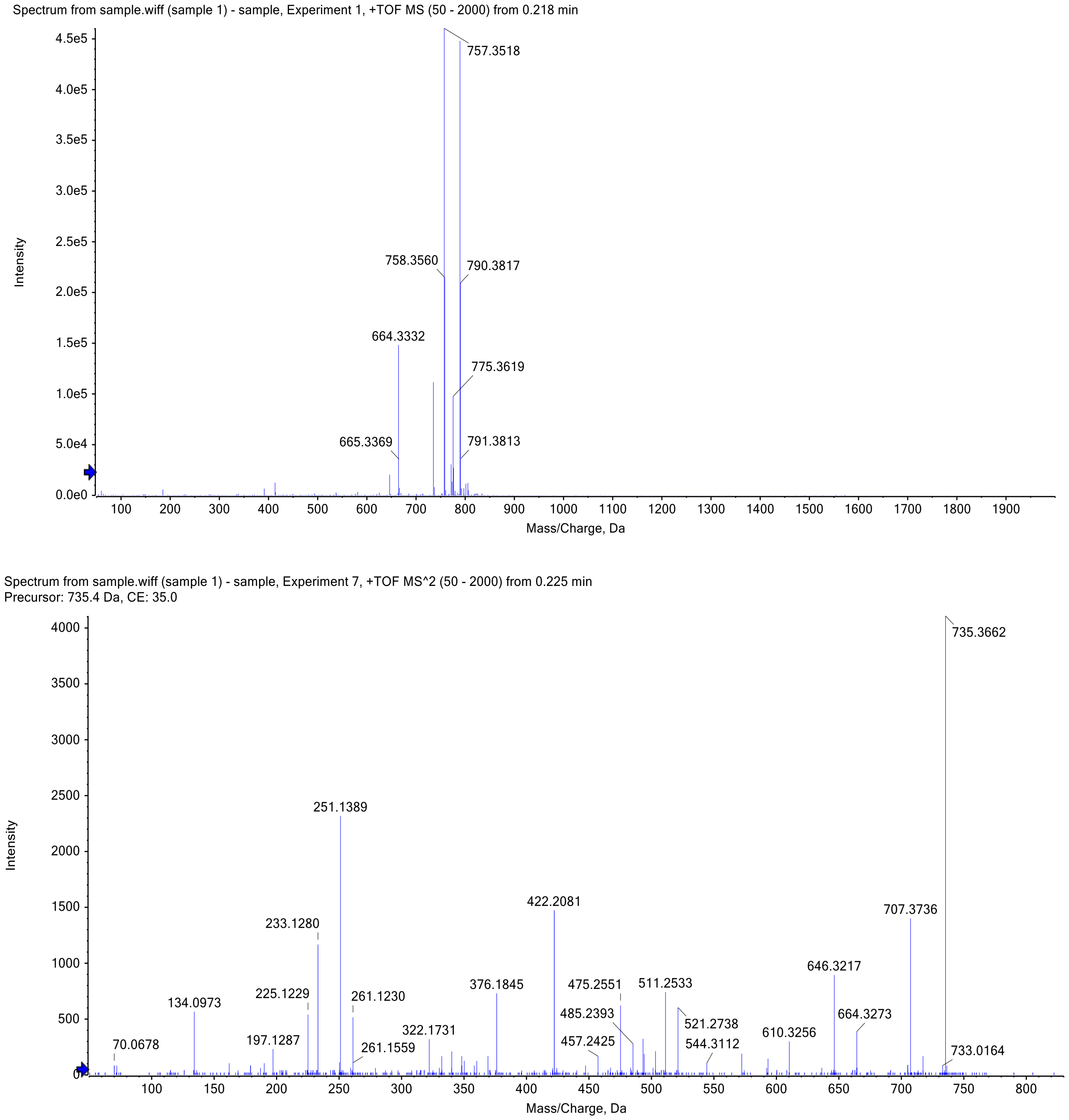

Figure S31. The high-resolution LC/MS-MS fragmentation analysis of FJ120DPA (**3**)

(a) JG002CPA (**1**)

L-FDAA

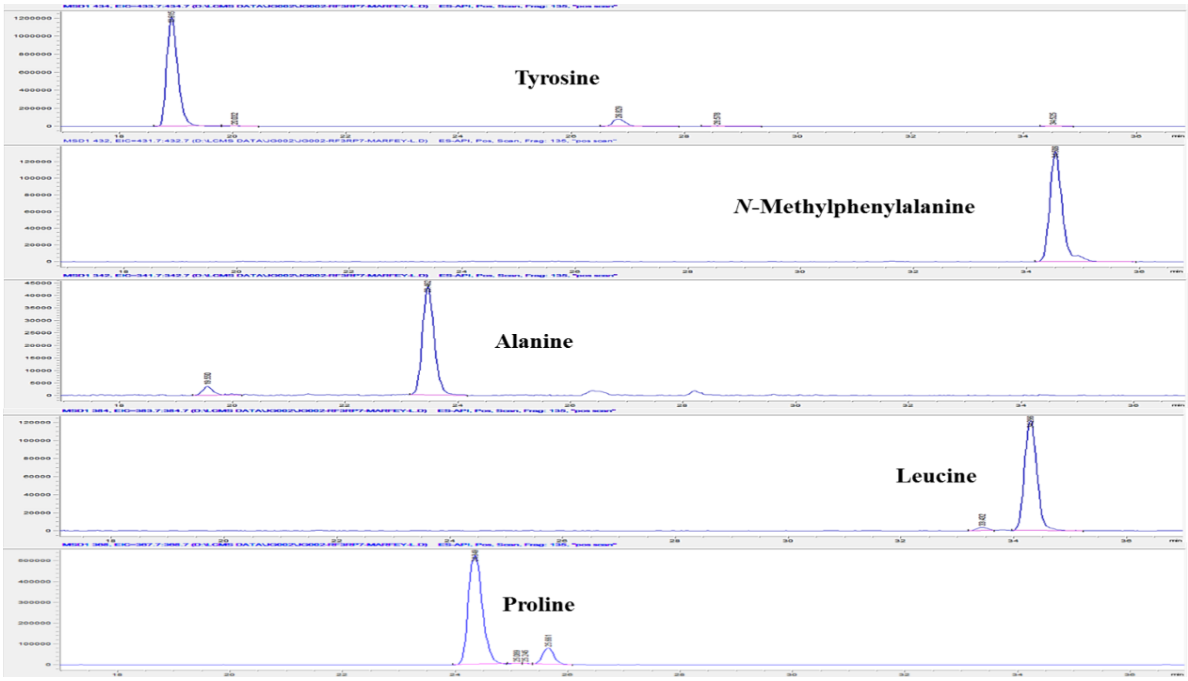

D-FDAA

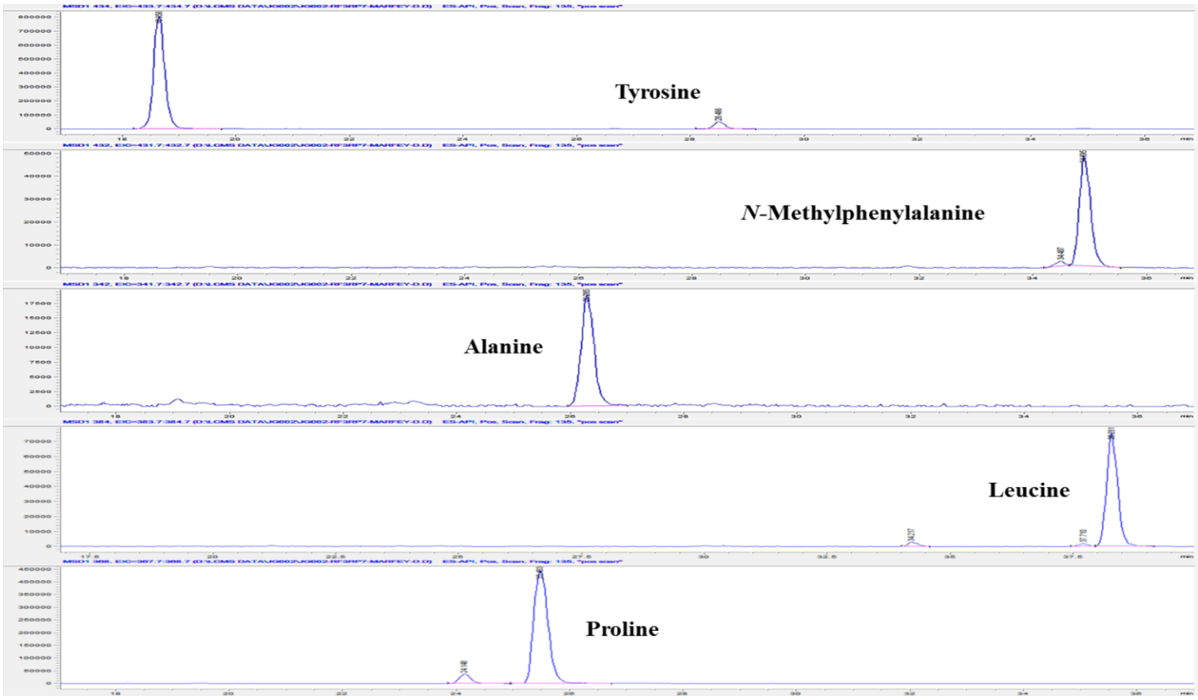

(b) JG002CPB (**2**)

L-FDAA

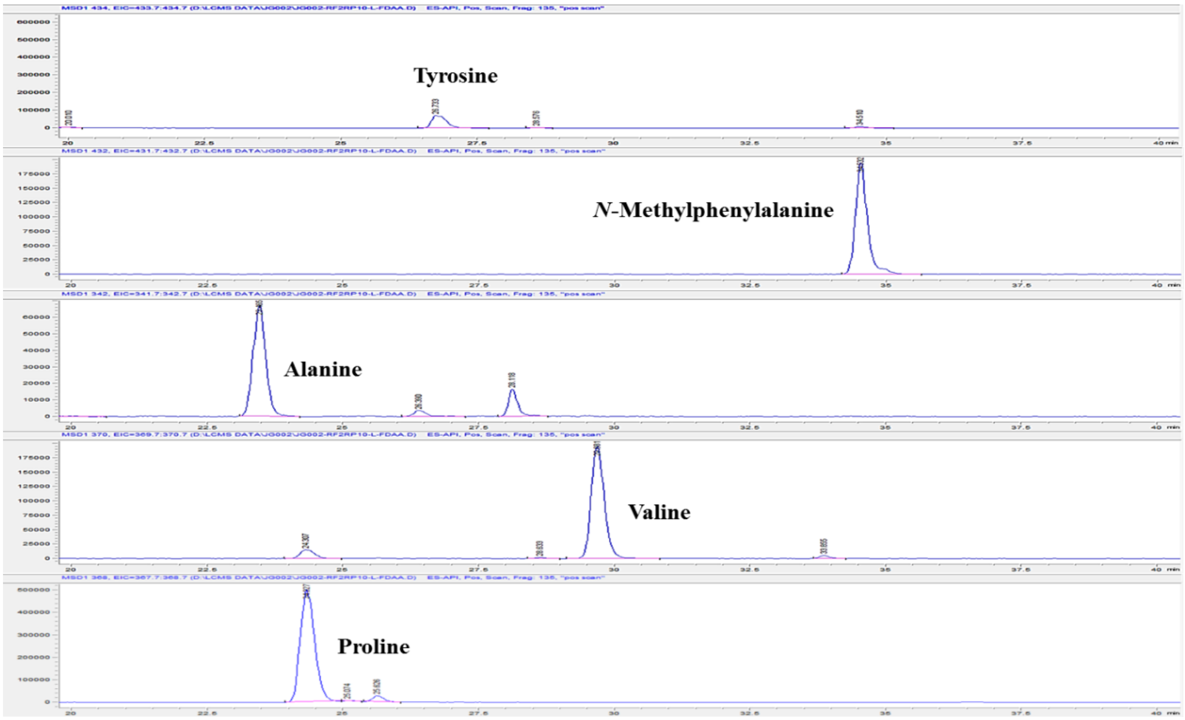

D-FDAA

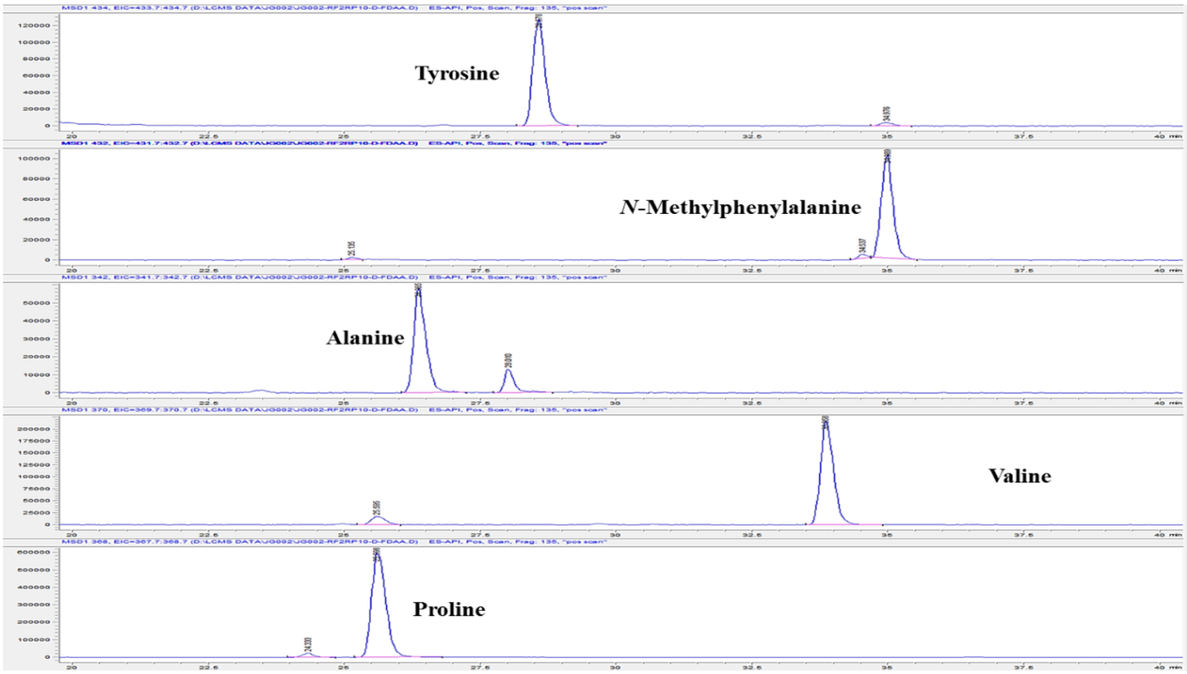

(c) FJ120DPA (**3**)

L-FDAA

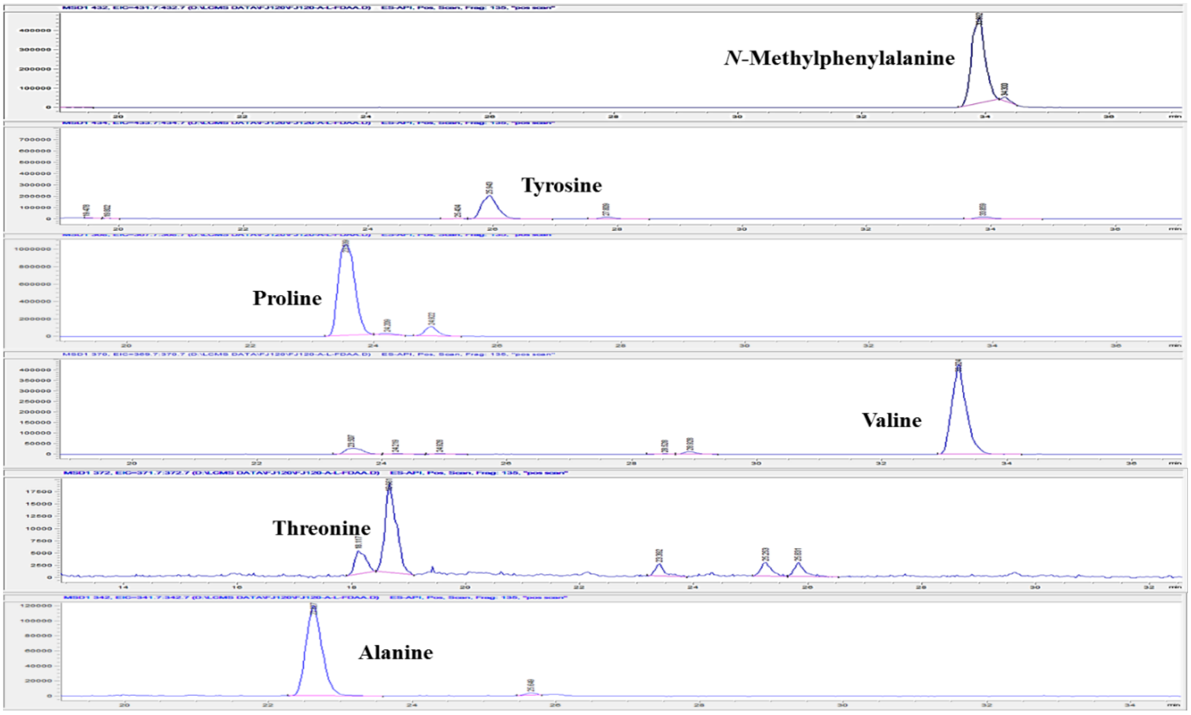

D-FDAA

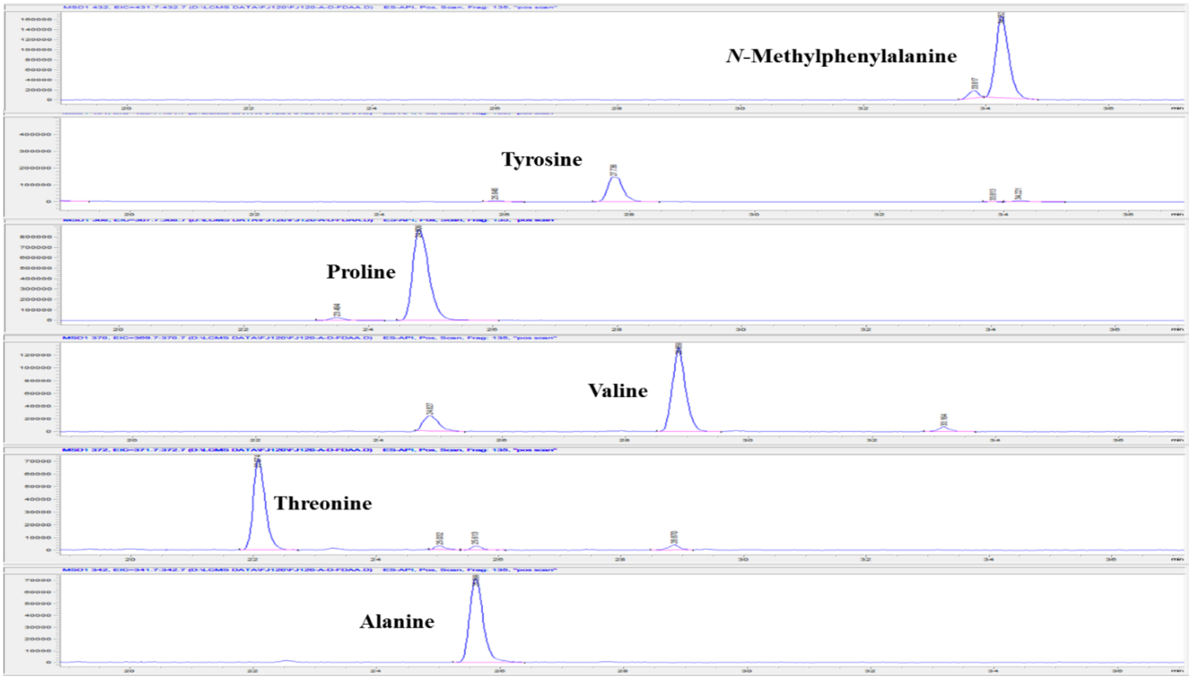

(d) FJ120DPB (**4**)

L-FDAA

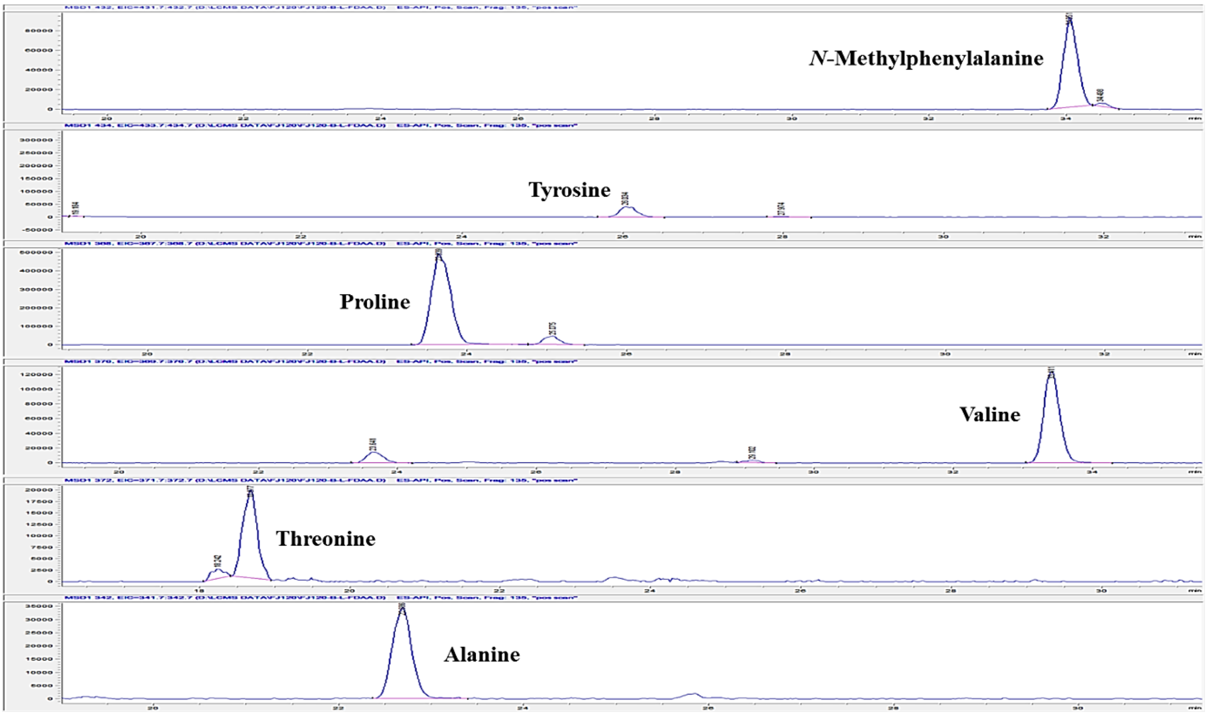

D-FDAA

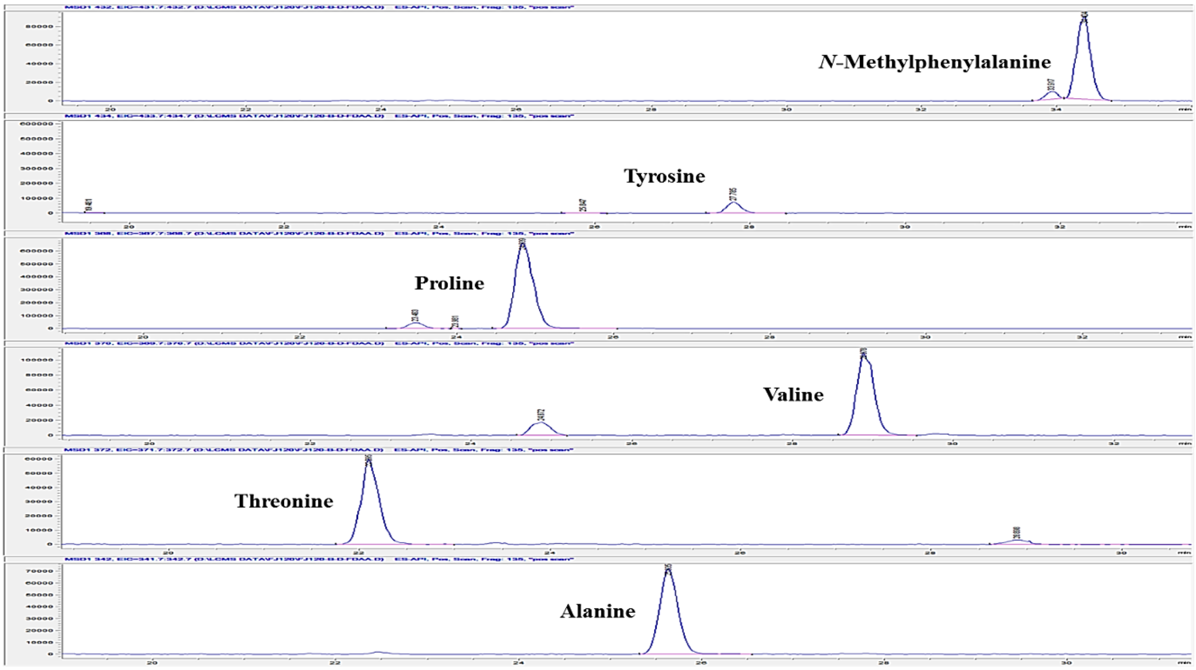

Figure S32. The LC analysis of L- and D-FDAA derivatives of the amino acid-derived units in compounds **1-4**

(a) JG002CPA (**1**)

**Amino acid**	t_{RL} (min)	t_{RD} (min)	Elution order	Δt (min)
**Tyrosine**	26.8	28.4	L → D	1.6
***N*-Methylphenylalanine**	34.4	34.9	L → D	0.5
**Alanine**	23.4	26.2	L → D	2.8
**Leucine**	34.3	38.2	L → D	3.9
**Proline**	24.3	25.4	L → D	1.1

(b) JG002CPB (**2**)

**Amino acid**	t_{RL} (min)	t_{RD} (min)	Elution order	Δt (min)
**Tyrosine**	26.8	28.5	L → D	1.7
***N*-Methylphenylalanine**	34.5	34.9	L → D	0.4
**Alanine**	23.4	26.4	L → D	3.0
**Valine**	29.6	33.8	L → D	4.2
**Proline**	24.3	25.6	L → D	1.3

(c) FJ120DPA (**3**)

**Amino acid**	t_{RL} (min)	t_{RD} (min)	Elution order	Δt (min)
**Tyrosine**	25.9	27.7	L → D	1.8
***N*-Methylphenylalanine**	33.8	34.3	L → D	0.5
**Alanine**	22.6	25.6	L → D	3.0
**Valine**	33.2	28.8	D → L	- 4.4
**Proline**	23.5	24.8	L → D	1.3
**Threonine**	18.6	22.1	L → D	3.5

(d) FJ120DPB (**4**)

**Amino acid**	t_{RL} (min)	t_{RD} (min)	Elution order	Δt (min)
**Tyrosine**	26.0	27.8	L → D	1.8
***N*-Methylphenylalanine**	34.0	34.4	L → D	0.4
**Alanine**	22.7	25.6	L → D	2.9
**Valine**	33.4	28.9	D → L	- 4.5
**Proline**	23.6	24.8	L → D	1.2
**Threonine**	18.7	22.1	L → D	3.4

$\Delta t = t_{RD} - t_{RL}$

Table S1. The LC/MS analysis of L- and D-FDAA derivatives of the amino acid-derived units in compounds **1-4**

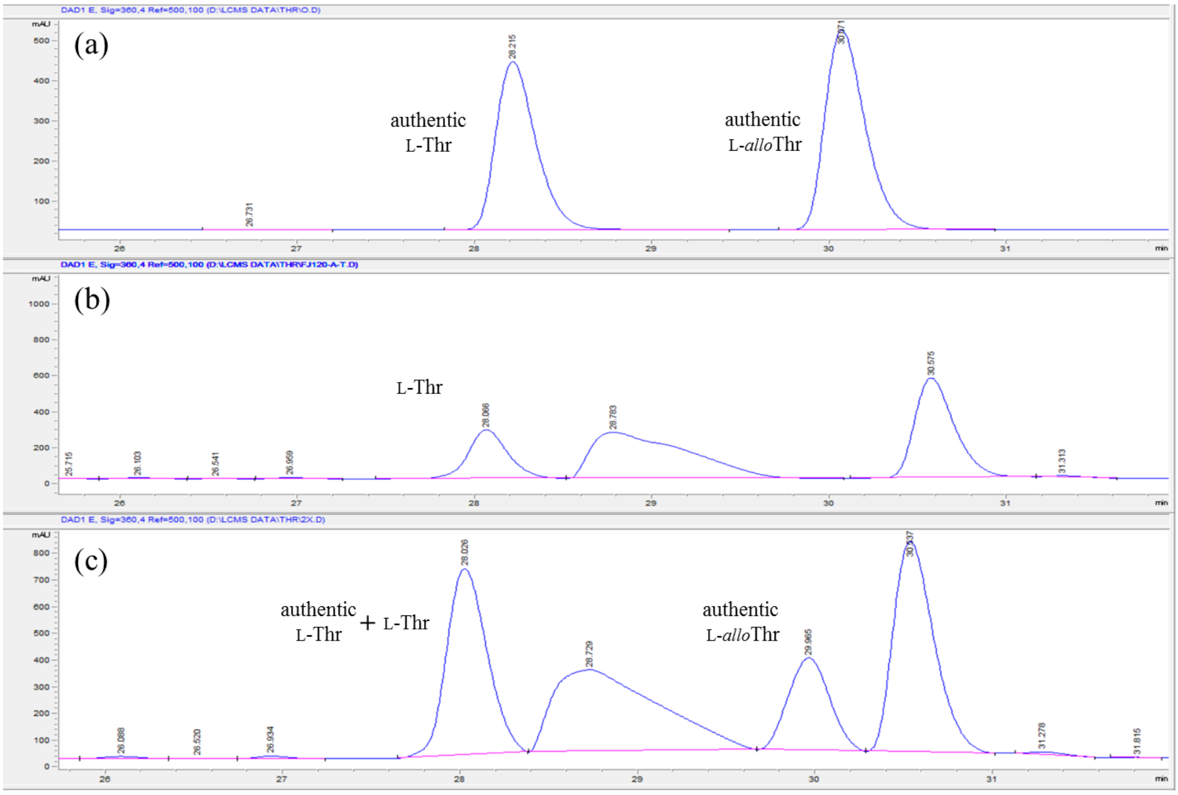

Figure S33. The LC analysis of L-FDAA derivatives of Thr and *allo*-Thr for FJ120DPA (**3**): (a) authentic L-Thr and L-*allo*-Thr, (b) L-Thr from **3**, (c) Co-injection of authentic L-Thr and L-*allo*-Thr with L-Thr from **3**

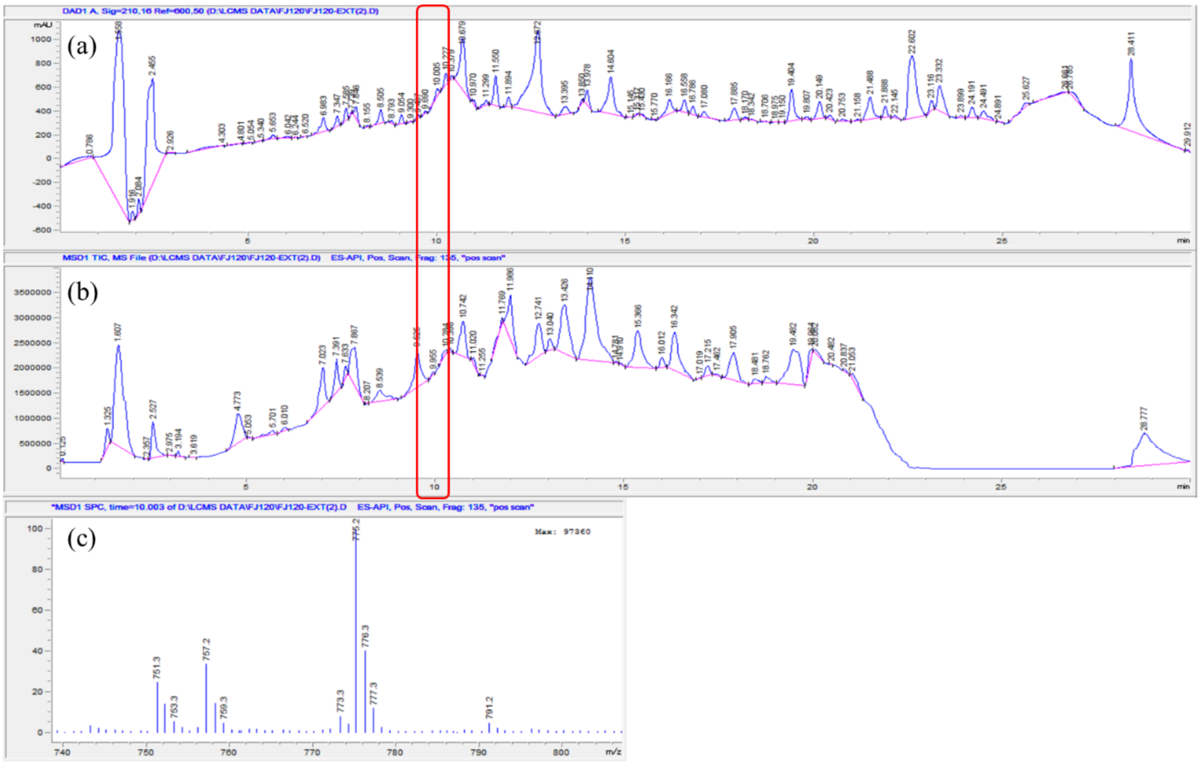

Figure S34. The LC-MS analysis of FJ120: (a) LC-UV of extract (b) ESI-LC-MS of extract (c) Positive ESI-MS of compound **4** at retention time of 10.003 in LC-ESI-MS spectrum.

			1		
**unit**	**position**	**δC, type**	**δH, mult (*J* in Hz)**	**COSY**	**HMBC**
Ala	CO	171,2, C			
	α	44.2, CH	3.54, dd (7.9, 6.8)	*β*, NH	CO, *β*
	β	17.1, CH_3	0.70, d (6.4)	*α*	CO, *α*
	NH		8.29, d (8.4)	*α*	*α*, COLeu
N-Me-Phe	CO	168.1, C			
	α	61.7, CH	4.17, dd (11.6, 3.6)	*β*	CO, *γ*, COAla
	β	33.9, CH_2	3.24, dd (14.3, 3.3)	*α*	CO, *γ*, *ortho*
			2.70, dd (14.4, 11.8)		
	γ	137.5, C			
	ortho	129.0, CH	7.09, d (7.2)	*meta*, *para*	*ortho*, *meta*, *para*
	meta	128.5, CH	7.28, d (7.6)	*ortho*, *para*	*γ*, *meta*
	para	126.7, CH	7.22, t (7.4)	*ortho*, *meta*	*ortho*
	N-CH_3	30.5, CH_3	2.61, s		*α*, COAla
Tyr	CO	168.9, C			
	α	52.6, CH	4.78, td (8.5, 5.2)	*β*, NH	CO, *β*
	β	37.3, CH_2	3.04, dd (13.4, 8.8)	*α*	CO, *α*, *γ*
			2.73, dd (13.5, 5.7)		
	γ	127.4, C			
	ortho	130.2, CH	7.04, d (8.4)	*meta*	*β*, *ortho*, *para*
	meta	114.9, CH	6.66, d (8.4)	*ortho*	*γ*, *meta*
	para	155.7, C			
	OH		9.19, s		*meta*, *para*
	NH		7.27, m	*α*	CO*N*-MePhe
Pro	CO	170.6, C			
	α	60.9, CH	4.10, dd (7.9, 1.7)	*β*	CO, *β*, *γ*, *δ*
	β	31.5, CH_2	1.95, m	*α*, *γ*	CO, *α*, *δ*
			1.88, m		
	γ	21.4, CH_2	1.77, m	*β*, *δ*	*α*, *β*
			1.58, m		
	δ	46.4, CH_2	3.47, m	*γ*	*γ*
			3.37, m		
Leu	CO	170.4, C			
	α	53.3, CH	4.19, m	*β*, NH	CO, *β*, *γ*
	β	41.3, CH_2	1.37, m	*α*, *γ*	CO, *γ*
			1.34, m		
	γ	24.5, CH	1.37, m	*β*, *δ*	*α*, *β*
	δ	22.4, CH_3	0.88, d (6.1)	*γ*	*β*, *γ*, *δ*
		21.7, CH_3	0.80, d (6.2)		
	NH		6.96, d (9.0)	*α*	*α*, COPro

Table S2. The NMR data of JG002CPA (**1**) in DMSO-d_6

		2			
**unit**	**position**	**δ_C, type**	**δ_H, mult (*J* in Hz)**	**COSY**	**HMBC**
Ala	CO	171.2, C			
	α	43.9, CH	3.60, dd (8.0, 6.8)	*β*, NH	CO, *β*
	β	17.1 CH_3	0.71, d (6.4)	*α*	CO, *α*
	NH		8.32, d (8.3)	*α*	*α*, COLeu
N-Me-Phe	CO	168.2, C			
	α	61.6, CH	4.16, dd (11.4, 3.4)	*β*	CO, *β*, COAla
	β	33.9, CH_2	3.25, dd (14.3, 3.3)	*α*	*α*, *γ*, *ortho*
			2.72, dd (14.4, 11.6)		
	γ	137.5, C			
	ortho	129.0, CH	7.10, d (7.2)	*meta*, *para*	*ortho*, *meta*, *para*
	meta	128.5, CH	7.28, d (7.6)	*ortho*, *para*	*γ*, *meta*
	para	126.7, CH	7.22, t (7.4)	*ortho*, *meta*	*ortho*
	N-CH_3	30.5, CH_3	2.63, s		*α*, COAla
Tyr	CO	168.9, C			
	α	52.7, CH	4.76, td (8.5, 5.2)	*β*, NH	CO, *β*, CO*N*-MePhe
	β	37.3, CH_2	3.02, dd (13.4, 9.0)	*α*	CO, *α*, *γ*, *ortho*
			2.74, dd (13.5, 4.7)		
	γ	127.4, C			
	ortho	130.2, CH	7.04, d (8.4)	*meta*	*β*, *ortho*, *meta*, *para*
	meta	114.9, CH	6.66, d (8.4)	*ortho*	*γ*, *meta*, *para*
	para	155.8, C			
	OH		9.21, s		*meta*, *para*
	NH		7.26, m	*α*	*α*, CO*N*-MePhe
Pro	CO	170.6, C			
	α	60.9, CH	4.10, dd (7.3, 2.6)	*β*	CO, *β*, *γ*, *δ*
	β	31.5, CH_2	1.93, m	*α*, *γ*	CO, *α*, *δ*
	γ	21.5, CH_2	1.78, m	*β*, *δ*	*α*, *β*
			1.61, dq (12.3, 9.1)		
	δ	46.2, CH_2	3.49, m	*γ*	*γ*
			3.35, m		
Val	CO	169.2, C			
	α	61.0, CH	3.82, t (9.6)	*β*, NH	CO, *β*, *γ*, COPro
	β	30.7, CH	1.69, m	*α*, *γ*	*α*, *γ*
	γ	19.3, CH_3	0.80, d (6.6)	*β*	*α*, *β*
		18.9, CH_3	0.78, d (6.7)		
	NH		7.00, d (8.7)	*α*	COPro

Table S3. The NMR data of JG002CPB (**2**) in DMSO-d_6

3					
**unit**	**position**	**δ_C, type**	**δ_H, mult (*J* in Hz)**	**COSY**	**HMBC**
Val	CO	172.0, C			
	α	57.1, CH	4.43, t (9.8)	*β*, NH	CO, *β*, *γ*, CO*N*-AcThr
	β	30.3, CH	1.89, m	*α*, *γ*	*α*, *γ*
	γ	19.2, CH_3	0.86, d (6.5)	*β*	*α*, *β*
		18.8, CH_3	0.92, d (6.5)		
	NH		6.43, d (9.1)	*α*	CO*N*-AcThr
Pro	CO	173.3, C			
	α	59.5, CH	4.31, d (5.8)	*β*	
	β	29.7, CH_2	2.13, m	*α*, *γ*	*γ*
	γ	24.7, CH_2	1.88, m	*β*, *δ*	*α*, *β*
	δ	48.1, CH_2	3.93, m	*γ*	*β*, *γ*
			3.52, m		
Tyr	CO	172.4, C			
	α	50.6, CH	4.54, dd (13.3, 6.6)	*β*, NH	*β*
	β	35.9, CH_2	2.42, dd (13.6, 9.2)	*α*	CO, *α*, *γ*, *ortho*
			1.87, dd (13.2, 6.2)		
	γ	126.6, C			
	ortho	130.2, CH	6.77, d (7.8)	*meta*	*ortho*, *para*
	meta	115.9, CH	6.66, d (8.0)	*ortho*	*γ*, *meta*, *para*
	para	155.9, C			
	NH		6.44, d (9.1)	*α*	
N-Me-Phe	CO	168.1, C			
	α	63.2, CH	4.82, t (6.8)	*β*	CO, *γ*, COTyr
	β	34.5, CH_2	3.34, dd (14.0, 5.7)	*α*	CO, *α*, *γ*, *ortho*
			2.52, dd (13.9, 7.8)		
	γ	137.9, C			
	ortho	129.4, CH	7.13, d (7.4)	*meta*, *para*	*β*, *ortho*, *para*
	meta	129.1, CH	7.24, d (7.5)	*ortho*, *para*	*γ*, *meta*
	para	127.3, CH	7.23, d (7.3)	*ortho*, *meta*	*ortho*
	N-CH_3	29.5, CH_3	2.87, s		*α*, COTyr
Ala	CO	170.9, C			
	α	48.5, CH	4.67, d (7.9)	*β*, NH	*β*
	β	18.6, CH_3	1.32, d (7.3)	*α*	CO, *α*
	NH		8.16, d (8.3)	*α*	*α*, CO*N*-MePhe
N-Ac-Thr	CO	169.7, C			
	α	55.4, CH	4.69, m	*β*, NH	CO, CO*N*-Ac
	β	70.1, CH	5.53, q (5.9)	*α*, *γ*	COAla
	γ	17.0, CH_3	1.18, d (6.5)	*β*	*α*, *β*
	NH		6.29, d (9.0)	*α*	CO*N*-Ac
	CO*N*-Ac	171.8, C			
	α	23.1, CH_3	1.83, s		CO*N*-Ac

Table S4. The NMR data of FJ120DPA (**3**) in $CDCl_3$

			4		
**unit**	**position**	**δ$_C$, type**	**δ$_H$, mult (*J* in Hz)**	**COSY**	**HMBC**
Val	CO	172.8, C			
	α	59.4, CH	4.10, d (9.0)	*β*	CO, *β*, *γ*
	β	30.8, CH	1.97, m	*α*, *γ*	*α*, *γ*
	γ	19.4, CH_3	1.02, d (6.6)	*β*	*α*, *β*
		19.2, CH_3	0.91, d (6.8)		
Pro	CO	173.7, C			
	α	61.7, CH	4.35, dd (8.3, 2.4)	*β*	CO, COVal
	β	30.5, CH_2	1.88, m	*α*, *γ*	CO, *α*, *γ*, *δ*
			1.74, m		
	γ	24.2, CH_2	1.63, m	*β*, *δ*	*α*, *β*
			1.04, m		
	δ	48.6, CH_2	3.72, m	*γ*	*β*, *γ*
			3.46, m		
Tyr	CO	173.9, C			
	α	51.2, CH	4.62, dd (11.2, 4.0)	*β*	CO, *β*
	β	36.0, CH_2	2.63, dd (13.1, 11.5)	*α*	CO, *α*, *γ*, *ortho*
			1.43, dd (13.2, 3.5)		
	γ	129.2, C			
	ortho	131.5, CH	6.92, d (8.3)	*meta*	*ortho*, *para*
	meta	115.9, CH	6.64, d (8.4)	*ortho*	*γ*, *meta*, *para*
	para	157.3, C			
N-Me-Phe	CO	170.5, C			
	α	63.7, CH	5.28, dd (10.9, 3.4)	*β*	CO, *β*, COTyr
	β	35.4, CH_2	3.16, dd (14.3, 3.5)	*α*	CO, *α*, *γ*
			2.97, dd (14.1, 9.3)		
	γ	139.4, C			
	ortho	130.7, CH	7.28, d (7.2)	*meta*, *para*	*β*, *ortho*, *para*
	meta	130.1, CH	7.30, d (7.3)	*ortho*, *para*	*γ*, *meta*
	para	128.1, CH	7.17, d (7.5)	*ortho*, *meta*	*ortho*
	N-CH_3	30.2, CH_3	2.90, s		*α*, COTyr
Ala	CO	180.8, C			
	α	52.1, CH	4.19, m	*β*	CO, *β*, CO*N*-MePhe
	β	18.5, CH_3	1.34, d (6.5)	*α*	CO, *α*
N-Ac-Thr	CO	173.0, C			
	α	59.7, CH	4.47, d (5.2)	*β*	CO, CO*N*-Ac
	β	68.6, CH	4.17, q (5.9)	*α*, *γ*	CO, *α*
	γ	19.7, CH_3	1.21, d (6.4)	*β*	*α*, *β*
	CO*N*-Ac	174.1, C			
	α	22.8, CH_3	2.09, s		CO*N*-Ac

Table S5. The NMR data of FJ120DPB (**4**) in CD_3OD
